# Allosteric Activation through Coordinated Energy Landscape Reweighting and Information Flow

**DOI:** 10.34133/csbj.0133

**Published:** 2026-06-09

**Authors:** Bao-Dan Zhang, De-Rui Zhao, Meng-Ting Liu, Li-Quan Yang, Peng Sang

**Affiliations:** ^1^ College of Agriculture and Biological Science, Dali University, Dali 671000, China.; ^2^ Key Laboratory of Bioinformatics and Computational Biology of the Department of Education of Yunnan Province, Dali University, Dali 671000, China.; ^3^ Co-Innovation Center for Cangshan Mountain and Erhai Lake Integrated Protection and Green Development of Yunnan Province, Dali University, Dali 671000, China.

## Abstract

Allosteric activation is commonly depicted as a ligand-driven transition between discrete structural states, yet such descriptions fail to explain how regulatory signals are dynamically organized and transmitted across protein architectures. In the innate immune adaptor Stimulator of interferon genes (STING), structural studies have resolved closed and open conformations of the ligand-binding domain, but how ligand binding reshapes the continuous conformational ensemble and its internal communication network remains unclear. Here, we integrate pathway-level representations of conformational change with extensive unbiased molecular dynamics and data-driven inference of interaction networks to examine STING activation beyond a binary structural switch. Binding of the cyclic dinucleotide C-di-GMP selectively reweights the conformational energy landscape, suppressing highly expanded states while stabilizing intermediate activation-prone conformations. Concomitantly, ligand binding reorganizes long-range allosteric communication by condensing dispersed, interface-dependent signal routes into shorter and predominantly intrachain pathways, thereby focusing information flow and enhancing transmission efficiency. Together, these results support a model in which STING activation is not adequately described as a discrete structural switch, but is associated with ligand-induced reorganization of interaction architecture, focusing of internal information flow, and reweighting of conformational energetics and kinetics within a physically accessible ensemble.

## Introduction

Allosteric regulation is traditionally interpreted through the lens of discrete structural transitions [1,2], in which ligand binding shifts a protein between well-defined inactive and active conformations. This view, rooted in static structural biology, has provided a powerful vocabulary for describing regulatory states and has been reinforced by an expanding catalogue of high-resolution structures. Yet, this apparent success conceals a fundamental limitation: structural state assignments alone do not specify how regulatory signals are dynamically organized, propagated [3], or selected from the vast space of motions accessible to proteins. Proteins populate continuous conformational ensembles spanning multiple timescales, and regulatory events often manifest not as rigid state switches but as redistributions of probability, kinetics, and internal communication [4,5]. Consistent with this ensemble-based view, recent computational frameworks have increasingly treated allostery as a residue-level communication problem, in which molecular dynamics (MD)-derived interaction networks and residue-specific nanoenvironment features can reveal allosteric pathways, hubs, and regulatory sites [6,7]. If activation is not governed by a simple conformational switch, a central unresolved question emerges: what physical principle determines which motions within a heterogeneous ensemble become functionally productive upon ligand binding? Addressing this question requires moving beyond endpoint structures toward a framework that treats allosteric activation as a coupled reorganization of energy landscapes and dynamic communication architectures, rather than as a transition between static forms [8–11].

**Fig. 1.**
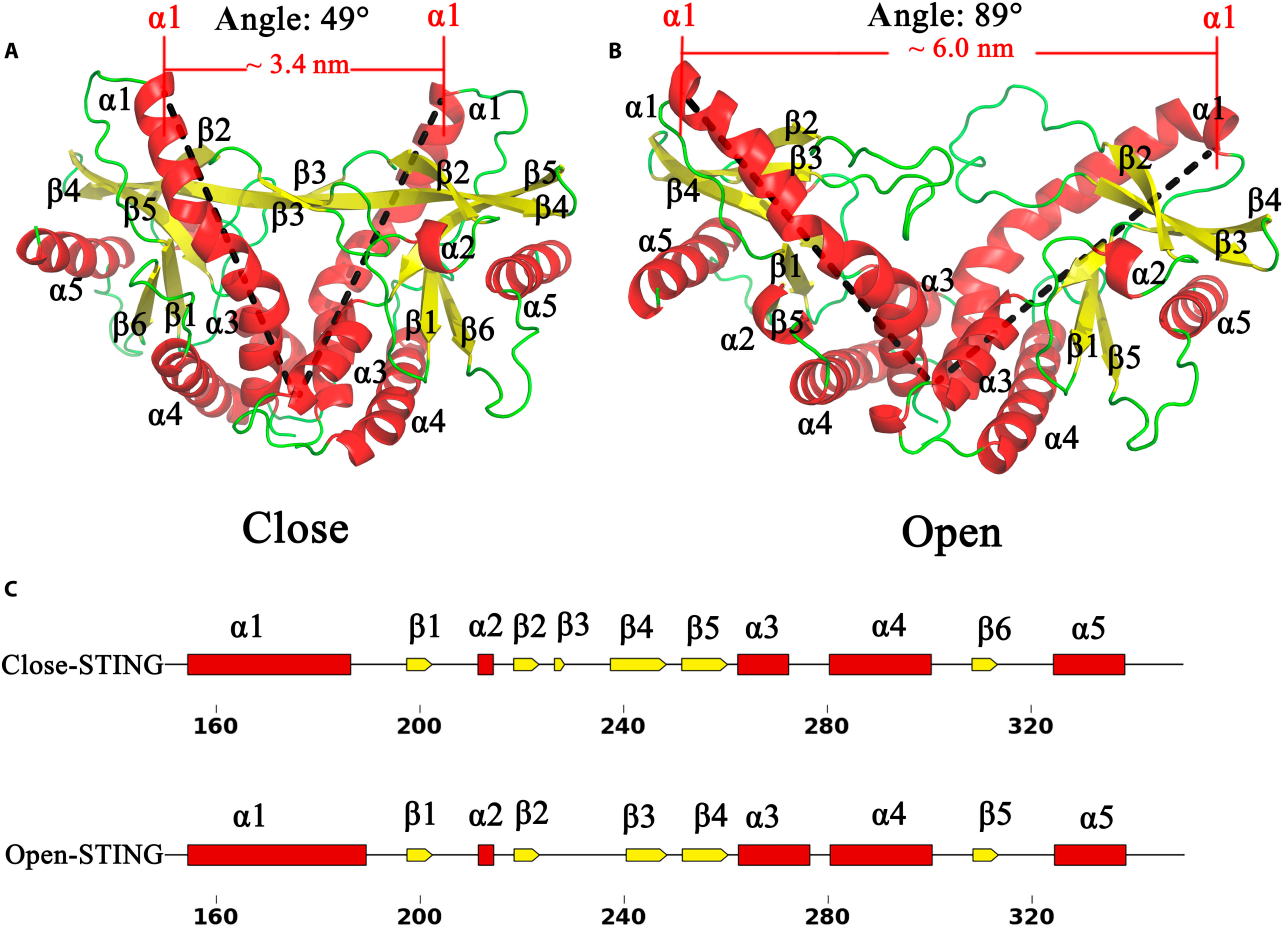
Large-amplitude, cooperative geometric reorganization underlies the closed–open transition of the STING ligand-binding domain. (A) Closed conformation of the STING ligand-binding domain (LBD) dimer, illustrating the compact geometry of the α1 helices at the dimer interface. In this state, the relative orientation and separation of the α1 helices define a tightly associated architecture characteristic of the inactive conformation, with an interhelical angle of ~49° and an end-to-end distance of ~3.4 nm. (B) Open conformation of the STING LBD dimer, revealing a pronounced and highly coordinated geometric reorganization at the interface. Relative to the closed state, the α1 helices undergo substantial rotational displacement and spatial separation, with the interhelical angle increasing to ~89° and the end-to-end distance expanding to ~6.0 nm. This large-scale motion spans several nanometers and reflects collective rearrangement of preformed structural elements rather than a simple local adjustment, indicating access to a broad and continuous conformational space during activation. (C) Comparison of secondary structure organization in the closed and open conformations of the STING LBD. Despite extensive global rearrangement at the dimer interface, the overall distribution of α-helices and β-sheets remains largely conserved between the 2 states. This conservation demonstrates that the closed–open transition is mediated primarily by relative motions among stable secondary structure elements, rather than by local unfolding or secondary structure remodeling.

The innate immune adaptor Stimulator of interferon genes (STING) [12–14] provides a particularly stringent system in which to interrogate these unresolved principles of allosteric activation. Structural studies have established that ligand binding to the STING ligand-binding domain (LBD) is accompanied by large-amplitude rearrangements at the dimer interface, most prominently involving changes in the relative separation and orientation of the α1 helices [15–17]. Notably, comparison of the experimentally resolved closed and open conformations reveals that this transition entails a highly coordinated geometric reorganization of the STING dimer, with the α1 helices separating by several nanometers and undergoing substantial rotational displacement (Fig. 1A and B). Such large-scale, cooperative motions already challenge a simple 2-state interpretation of STING activation, suggesting access to a broad and continuous conformational space rather than a discrete structural switch. Importantly, this extensive rearrangement occurs without wholesale remodeling of secondary structural elements, indicating that STING activation is mediated by relative motions among preformed structural units rather than local unfolding events (Fig. 1C). These observations expose a critical inconsistency: although structural endpoints of STING activation are well characterized, they provide no explanation for how ligand binding selectively biases the underlying conformational ensemble [9] or reorganizes long-range communication across the dimer to enable signaling [18].

Resolving this problem requires an approach that treats allosteric activation as an emergent, multiscale process rather than as a sequence of isolated structural events [18,19]. Capturing such behavior demands simultaneous access to 3 levels of description: the continuity of conformational transitions connecting experimentally resolved endpoints, the statistical redistribution of populations and timescales across the energy landscape, and the dynamic organization of long-range communication pathways that couple distal regions of the protein [20]. No single structural snapshot or conventional simulation protocol can satisfy these requirements in isolation. Instead, a coherent strategy must integrate pathway-level representations of conformational change with unbiased dynamicsampling and network-based inference of information flow [21,22]. By doing so, it becomes possible to ask not only which conformations are visited upon ligand binding, but how the probabilities, kinetics, and internal communication architectures are collectively reorganized to favor activation-competent states.

Here, we apply this integrative perspective to interrogate ligand-induced activation of the STING LBD as a problem of dynamic reorganization. Using experimentally resolved closed and open conformations as boundary conditions, we construct a continuous activation pathway that defines the structural manifold connecting inactive and active states. By initiating extensive, unbiased MD simulations from multiple points along this pathway, we systematically sample the conformational ensemble accessible to STING in both the apo and ligand-bound forms. We then combine statistical kinetic modeling with data-driven network inference to quantify how ligand binding redistributes conformational populations, reshapes transition kinetics, and reorganizes long-range communication pathways across the STING dimer. This strategy enables us to link changes in the energy landscape to corresponding rewiring of internal information flow, thereby providing a unified view of how ligand binding biases STING toward activation-competent dynamic regimes.

## Materials and Methods

### Defining a physically accessible conformational ensemble

To investigate how ligand binding reshapes STING activation beyond discrete structural endpoints, we first defined a conformational ensemble that is both continuous in geometry and physically accessible under realistic energetic constraints. Although experimentally resolved closed and open structures of the STING LBD represent 2 functional extremes, these static conformations alone do not specify how the protein traverses conformational space during activation, nor which intermediate configurations are dynamically relevant. We therefore sought to construct a structurally and physically grounded ensemble that spans the closed–open transition while preserving the intrinsic collective degrees of freedom of the STING LBD.

Experimentally determined closed and open conformations of the STING LBD were obtained from the Protein Data Bank (PDB entries 4F5D [23] and 4F5Y [24], respectively), both solved by x-ray crystallography. To ensure strict comparability across different conformations in subsequent dynamic analyses, a unified preprocessing procedure was applied. Specifically, only the LBD (residues 153 to 340 of chains A and B) was retained from both structures. This treatment ensured identical chain composition, residue numbering, and atom counts across all conformations, thereby eliminating systematic biases arising from structural mismatches. All subsequent analyses were based on these uniformly processed LBD structures.

Using the closed and open conformations as boundary conditions [25], the conformational transition pathway connecting these 2 states was reconstructed with the MinActionPath2 method [26–28]. This approach, grounded in the principle of minimum action, identifies the statistically most probable transition pathway in high-dimensional conformational space given defined initial and final states. Importantly, the resulting pathway does not represent a real-time MD trajectory; rather, it provides a continuous and physically meaningful structural reference that captures collective motions favored under equilibrium constraints and spans the closed–open transition of the STING LBD. We selected MinActionPath2 rather than constrained MD to generate the initial intermediate conformations for 2 main reasons. First, the purpose of this step was not to reconstruct a real-time activation trajectory, but to define a structurally continuous and physically plausible set of starting conformations spanning the experimentally observed closed and open endpoints. MinActionPath2 generates nonlinear transition paths by minimizing an action functional under a coarse-grained elastic-network-based framework, thereby providing intermediate structures that are guided by collective mechanical couplings between the 2 endpoint conformations. Second, constrained MD would require predefined reaction coordinates or collective variables to drive the closed-to-open transition. For the STING LBD, where activation involves coupled rotation and separation of dimeric structural elements, imposing such coordinates could bias the pathway toward the chosen constraints and potentially underrepresent alternative collective motions. We therefore used MinActionPath2 as an unbiased path-generation step to obtain representative initial conformations, which were subsequently subjected to independent, unbiased all-atom MD simulations for ensemble sampling and downstream Markov state model (MSM) and neural relational inference (NRI) analyses.

From the continuous minimum-action pathway, 8 representative intermediate conformations were selected at approximately uniform intervals along the path (path indices 4, 8, 15, 23, 30, 38, 46, and 54). These intermediates collectively capture the dominant geometric features of the transition while maintaining intact secondary structure elements. Together with the closed and open endpoint structures, this procedure yielded a total of 10 distinct conformational states, which define a connected but constrained conformational ensemble spanning the activation-relevant region of conformational space. Based on this ensemble, 2 classes of simulation systems were constructed: the apo STING LBD system and the STING LBD–C-di-GMP complex system.

For construction of the C-di-GMP-bound systems, the experimentally resolved ligand-bound structure was used whenever available. Specifically, for the 4F5D-derived conformation, the crystallographic C-di-GMP pose was retained directly and was not generated by molecular docking. For the MinActionPath2-derived intermediate conformations and other conformational states for which no corresponding experimental ligand-bound structures were available, C-di-GMP was placed into the STING LBD binding pocket using AutoDock Vina [29].

A cubic docking grid centered on the ligand-binding region was defined with dimensions of 40 × 40 × 40 Å^3^ and center coordinates of *x* = 37.17, *y* = −18.788, and *z* = 3.101.

To assess whether the docked ligand pose was consistent with the experimentally resolved binding mode, the protein backbone of the docked complex was aligned to the 4F5D ligand-bound structure, and the heavy-atom root-mean-square deviation (RMSD) between the docked C-di-GMP pose and the crystallographic C-di-GMP pose was calculated. The docked pose showed close agreement with the crystallographic pose, with a ligand heavy-atom RMSD of 0.830 Å. We also inspected the binding pocket and confirmed that the docked C-di-GMP remained located in the canonical ligand-binding site and preserved the overall orientation and major interaction features observed in the experimental structure. The superposition of the docked and crystallographic C-di-GMP poses and the binding-pocket comparison are provided in Fig. S1. These results support the use of the docked C-di-GMP poses as initial ligand-bound configurations for noncrystallographic intermediate and open-like STING LBD conformations.

Crucially, the same set of 10 initial conformations—comprising the closed state, the open state, and 8 intermediate states—was used for both the apo and ligand-bound systems. This design ensured systematic and comparable coverage of functionally relevant regions of conformational space and provided a unified structural foundation for subsequent unbiased MD simulations, ensemble reweighting analyses, and network-based investigations of ligand-induced allosteric regulation.

### Ensemble-based MD simulations

To interrogate how ligand binding reshapes STING activation at the level of conformational energetics and dynamics, we performed extensive unbiased MD simulations [30] designed to consistently sample the physically accessible conformational ensemble defined above. Rather than relying on a single initial structure, simulations were initiated from multiple conformations spanning the closed–open transition, enabling systematic assessment of how dynamic behavior depends on position within the ensemble and how this dependence is altered by ligand binding.

All MD simulations [21,30] were carried out using GROMACS 2023 [31]. The initial configurations comprised the closed conformation, the open conformation, and 8 intermediate states (state 1 to state 8) uniformly selected along the minimum-action pathway generated by MinActionPath2 [26,27], yielding a total of 10 representative conformations. For each initial conformation, 2 simulation systems were constructed: the apo STING LBD system and the STING LBD–C-di-GMP complex system.

For each system type, unbiased MD simulations of 1 μs were performed starting from each of the 10 conformations, resulting in 20 independent trajectories and an aggregate simulation time of 20 μs. This multistart design minimizes path dependence on any single structure and enables ensemble-consistent comparisons between the apo and ligand-bound states.

Protein dynamics were described using the CHARMM36 force field [32,33], which has been extensively validated for protein conformational dynamics. For the STING–C-di-GMP complex, force-field parameters for C-di-GMP were generated using the CHARMM General Force Field (CGenFF) [34] through the ParamChem program, using CGenFF program version 4.0 compatible with CGenFF version 4.6. Parameter quality was evaluated from the CGenFF penalty scores in the generated stream (.str) file. The maximum parameter and charge penalties were 27.0 and 17.901, respectively, both within the moderate penalty range of 10 to 50, and no penalty exceeded 50. The highest penalty corresponded to a dihedral term near the sugar–base linkage. We further inspected the MD trajectories and confirmed that the ligand retained a stable binding conformation without abnormal torsional distortion in this region. These results indicate that the CGenFF-derived parameters are acceptable for the present simulations. To further assess the stability of the docked ligand pose, ligand heavy-atom RMSD was calculated for 3 representative STING–C-di-GMP complex trajectories initiated from the closed conformation, one intermediate conformation, and the open conformation. For each trajectory, RMSD was calculated after least-squares alignment to the protein backbone, using the initial ligand-bound structure of the corresponding trajectory as the reference. This analysis evaluates the positional stability of C-di-GMP relative to the STING LBD binding pocket. No positional restraints were applied to C-di-GMP during production simulations.

Each system was placed in a suitably sized dodecahedral periodic simulation box and solvated explicitly with the TIP3P water model [35]. Counterions (Na^+^ and Cl^−^) were added to neutralize the system and to achieve a physiological ionic strength of 0.15 M [36]. Energy minimization [37] was performed using the steepest descent algorithm to eliminate unfavorable contacts. Systems were then equilibrated sequentially under constant volume and temperature (NVT) and constant pressure and temperature (NPT) ensembles for 100 ps each [38], during which positional restraints [39] were applied to protein heavy atoms to allow solvent relaxation while preserving the initial protein geometry.

Production MD simulations were conducted under periodic boundary conditions at 310 K [40], with temperature regulated using a velocity-rescaling thermostat and pressure maintained at 1 bar using the Parrinello–Rahman [41] barostat. Long-range electrostatic interactions were treated using the particle mesh Ewald method [42], with a nonbonded cutoff of 1.0 nm. All covalent bonds involving hydrogen atoms were constrained using the LINCS algorithm [39], permitting a time step of 2 fs. Trajectory frames were saved every 10 ps for subsequent analyses.

By performing multistart, microsecond-scale unbiased MD simulations across a structurally continuous ensemble, this strategy systematically samples the thermodynamically accessible conformational space of the STING LBD without imposing external biases or restraints. Importantly, this ensemble-consistent sampling framework enables direct comparison of dynamic behavior across corresponding regions of conformational space in the apo and ligand-bound systems, providing a robust foundation for subsequent analyses of fluctuation redistribution, free-energy landscape reweighting, kinetic organization, and ligand-induced allosteric network reprogramming.

### State-resolved thermodynamic and kinetic reconstruction of the conformational ensemble

To resolve how ligand binding reorganizes the conformational ensemble of the STING LBD at the level of thermodynamic stability and transition kinetics, we constructed MSMs [43,44] based on the multistart, microsecond-scale unbiased MD trajectories described above. Rather than treating conformational fluctuations as continuous noise, MSMs provide a state-resolved representation in which conformational space is discretized into metastable states connected by kinetically meaningful transitions [45], enabling quantitative characterization of equilibrium populations and interconversion dynamics.

The MSM framework is particularly well suited to the present study because conformational activation of STING unfolds across multiple timescales and involves transitions that are not fully captured within any single trajectory. By integrating information from multiple independent simulations initiated from distinct regions of conformational space, MSMs reconstruct long-timescale dynamics under the assumption that, at a sufficiently long lag time, the system’s future evolution depends only on its current state. This ensemble-based formulation enables direct comparison of thermodynamic and kinetic organization between the apo and ligand-bound systems under identical sampling protocols.

MSMs were constructed separately for the apo STING LBD system and the STING LBD–C-di-GMP complex system using the PyEMMA [46] software package (version 2.5.10). For each system, the trajectory dataset comprised 10 independent 1-μs simulations initiated from the closed conformation, the open conformation, and 8 intermediate states spanning the minimum-action pathway, yielding a total of 10 μs of unbiased sampling per system [47]. This symmetric, multistart design ensures comparable statistical coverage of conformational space and minimizes bias arising from initial structure selection.

To capture the dominant slow degrees of freedom associated with STING activation, MSM input features were chosen to explicitly reflect large-scale conformational rearrangements of the LBD dimer. Specifically, Cα–Cα distances between corresponding residues in the β2 strands (residues 219 to 224) of chains A and B were computed for all trajectory frames, providing a compact and physically interpretable descriptor of relative subunit separation and reorientation [48]. The resulting high-dimensional feature space was projected onto a low-dimensional kinetic subspace using time-lagged independent component analysis (TICA) [49], which isolates collective motions dominating slow conformational dynamics. Only the leading independent components (ICs) governing slow processes were retained for subsequent state discretization.

In the TICA-projected space, conformational space was discretized into 1,000 microstates using k-means clustering. This number of microstates was chosen to provide sufficient resolution of the activation-relevant conformational landscape while maintaining adequate transition statistics within the available sampling. Transition probability matrices were estimated at a lag time of 5.5 ns. This lag time was selected based on implied timescale (ITS) analysis [50], which was used to evaluate the convergence of the dominant slow processes as a function of lag time. The ITS profiles showed that the leading slow timescales reached an approximately stable regime beyond ~5 ns; therefore, 5.5 ns was selected as the lag time for MSM construction.

To further evaluate model robustness, ITS analysis was performed with uncertainty estimates from Bayesian MSM posterior sampling, shown in Fig. S2. Markovian behavior was further assessed using Chapman–Kolmogorov tests [20] at the macrostate level, comparing MSM-predicted transition probabilities with direct estimates from the simulation data (Fig. S3). For mechanistic interpretation, microstates were coarse-grained into 3 metastable macrostates using Perron cluster analysis (PCCA+) [51], enabling comparison of dominant conformational basins and their kinetic connectivity between the apo and C-di-GMP-bound systems.

Macrostate populations, free-energy differences, and mean first passage times (MFPTs) were estimated from the Bayesian posterior ensemble of MSM transition matrices. Unless otherwise stated, reported macrostate populations and transition times correspond to posterior mean or median estimates, with uncertainty quantified by 95% credible intervals. Posterior distributions of MFPTs and macrostate populations are provided in Figs. S4 and S5, respectively.

As a feature-robustness control, an additional MSM was constructed using interchain interface contact features between chains A and B, defined with distance thresholds of 0.4 and 0.7 nm. This alternative model was analyzed using the same TICA, clustering, lag-time validation, and PCCA+ workflow as the primary β2-distance-based MSM.

By reconstructing the conformational ensemble in a state-resolved manner, MSM analysis enables discrimination between mere suppression of fluctuations and genuine reweighting of thermodynamic stability and kinetic accessibility. This framework allows determination of not only which conformations are sampled upon ligand binding, but also how their relative populations and transition pathways are reorganized, providing the quantitative backbone for linking ligand-induced dynamic redistribution to biased ensemble organization and activation-relevant kinetics in subsequent analyses.

### Dynamic inference of interaction architecture and information flow

To resolve how ligand binding reorganizes long-range coupling and information transmission within the STING LBD, we inferred dynamic interaction architectures directly from MD trajectories using an NRI [52,53] framework. Unlike static contact-based networks, this approach infers time-dependent dynamic dependencies that govern how motions at one site influence motions elsewhere, thereby providing a functional description of intraprotein information flow. In this formulation, residues are represented as nodes in a graph, and probabilistic edges encode latent interaction relationships that best explain the coordinated temporal evolution of residue motions, rather than instantaneous geometric proximity.

To ensure that inferred interaction patterns reflect ensemble-level organization rather than trajectory-specific artifacts, NRI analysis was performed on the same multistart, microsecond-scale unbiased MD dataset used for MSM construction. For both the apo and C-di-GMP-bound systems, 10 independent 1-μs trajectories initiated from distinct conformational states spanning the closed–open transition were analyzed, yielding 10 μs of sampling per condition. Each trajectory was treated as an independent realization of the underlying dynamic process, analogous to separate experimental replicates. Integrating information across trajectories initiated from diverse regions of conformational space minimizes path dependence and emphasizes robust dynamic couplings that persist across the ensemble.

To focus on collective and long-range coordination while maintaining computational tractability, a coarse-grained graph representation was adopted. One Cα atom was selected every 2 residues, resulting in 188 nodes representing the STING LBD [54]. Each node was described by its normalized 3-dimensional coordinates and velocities, jointly encoding structural configuration and dynamic evolution. This level of coarse-graining preserves the spatial organization of the protein while filtering out high-frequency local noise, thereby emphasizing cooperative motions relevant to allosteric communication.

The NRI model employs a standard encoder–decoder architecture, in which the encoder infers a latent interaction graph that best explains the observed temporal evolution of node states, and the decoder uses this graph to predict future dynamics. Model training therefore imposes a physical constraint: only interaction architectures that enable accurate dynamic prediction are retained, providing a natural regularization against spurious correlations. Trajectories were segmented into overlapping time windows of 50 frames, sampled with a stride of 100 frames, yielding a large ensemble of short dynamic sequences. The model was trained using the Adam optimizer with an initial learning rate of 5 × 10^−4^ for 500 epochs, and the optimal model was selected based on validation loss.

Following training, inferred edge probabilities were used to construct residue-level dynamic coupling networks for the apo and C-di-GMP-bound systems. The NRI model was trained using all 10 independent 1-μs trajectories for each condition, and the resulting networks therefore represent ensemble-level dynamic coupling architectures rather than single-trajectory interaction patterns. For the primary network analysis and visualization, inferred interaction graphs were thresholded at an edge probability of 0.2 to identify marked dynamic couplings. Based on these inferred couplings, residue-level and region-level interaction matrices were constructed to quantify changes in coupling strength upon C-di-GMP binding.

To assess whether the inferred coupling topology depended on the specific edge-probability cutoff, we further repeated the NRI network analysis using a more permissive threshold of 0.1. This threshold-sensitivity analysis was used to evaluate whether the qualitative apo-versus-bound differences in coupling organization were preserved under a relaxed definition of markeddynamic coupling.

In this study, the term “information flow” is used as a mechanistic shorthand for effective communication routes inferred from the NRI-derived dynamic coupling network. It does not refer to Shannon information or an explicit information-theoretic quantity. Instead, it denotes the preferential propagation of dynamic coupling through high-probability interaction paths inferred from trajectory-based predictive dependencies.

To characterize these effective communication routes, edge-weighted shortest-path analyses were performed on the NRI-inferred interaction architecture. Edge weights were defined as the inverse of inferred interaction strength, such that paths composed of stronger dynamic couplings were assigned lower effective costs. This formulation identifies communication routes that are both dynamically strong and topologically efficient, without imposing predefined signaling pathways. Through this procedure, information-flow routes emerge from the inferred dynamic interaction architecture rather than from predefined structural assumptions.

As an independent comparison with a conventional trajectory-based correlation method, dynamic cross-correlation matrices (DCCMs) were calculated from Cα atomic fluctuations after least-squares alignment of each trajectory to the STING LBD backbone. The normalized cross-correlation coefficient between residues *i* and *j* was calculated from their displacement vectors relative to their time-averaged positions. DCCMs were calculated separately for the apo and C-di-GMP-bound systems. Region-level DCCM networks were further constructed using the same 6-region partitioning scheme as in the NRI analysis, providing a conventional correlation-based reference for comparison with the NRI-inferred coupling architecture.

## Results

### A physically accessible conformational ensemble connects inactive and active states

To enable systematic dynamic sampling of STING activation, it is essential to define intermediate conformations that are not only geometrically intermediate between inactive and active structures, but also physically accessible under realistic energetic and mechanical constraints. Although the closed and open conformations of the STING LBD can, in principle, be connected by infinitely many continuous paths in conformational space, only a restricted subset should correspond to collective rearrangements compatible with the intrinsic couplings of the dimeric scaffold. We therefore sought to delineate a physically accessible conformational ensemble that specifies the dominant degrees of freedom underlying the closed–open transition and constrains the activation problem to motions the protein can realistically realize.

**Fig. 2.**
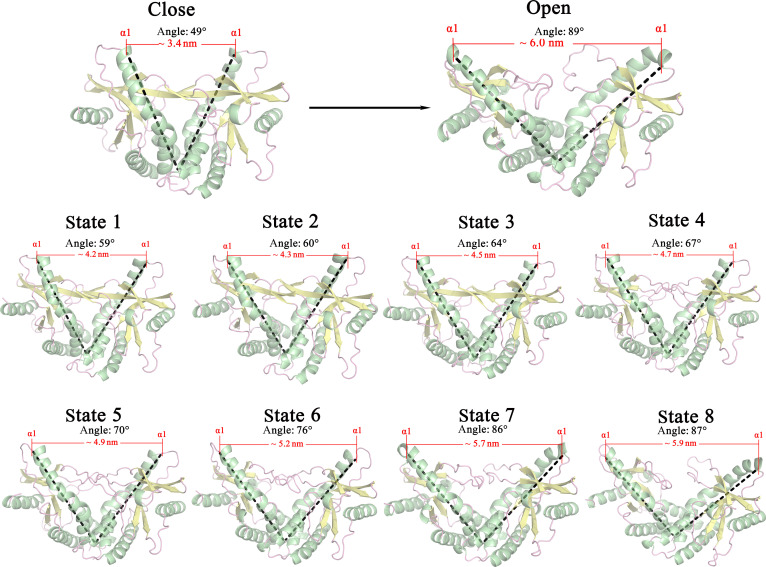
Continuous conformational transition pathway of the STING ligand-binding domain (LBD) connecting closed and open states. The conformational transition pathway of the STING LBD from the closed state (Close, PDB: 4F5D) to the open state (Open, PDB: 4F5Y) was constructed using the MinActionPath2 method, and 8 representative intermediate conformations (state 1 to state 8) were selected along the pathway. The STING LBD dimer is shown in cartoon representation, with the α1 helices colored green, β-sheet regions shown in yellow, and remaining regions shown in pink. Red annotations indicate geometric descriptors of the dimer interface, including the α1–α1 distance (nm) and interhelical angle (°), while black dashed lines highlight changes in the relative orientation of the α1 helices. Along the pathway, the α1–α1 distance increases continuously from approximately 3.4 nm in the closed state to approximately 6.0 nm in the open state, accompanied by a monotonic increase in the interhelical angle from ~49° to nearly 90°. These smooth and coordinated geometric changes indicate that the closed–open transition is dominated by collective reorientation and separation of preformed α1 helices, rather than by abrupt rearrangements or local unfolding events. The intermediate conformations shown here span the major geometrical features of the closed-to-open transition and define a physically accessible conformational ensemble. This ensemble serves as the structural basis for subsequent multistart molecular dynamics simulations and state-resolved analyses of ligand-induced dynamical reweighting and allosteric regulation.

Using the experimentally resolved closed (PDB: 4F5D) and open (PDB: 4F5Y) structures as boundary conditions, we reconstructed the most probable transition pathway with MinActionPath2. Eight representative intermediate conformations (state 1 to state 8) were selected along this pathway together with the 2 endpoints, yielding a set of structures that spans the full range of geometries involved in the transition (Fig. 2). Rather than serving as a time-ordered kinetic trajectory, this pathway provides a structural reference that captures collective rearrangements favored under equilibrium constraints and thus defines a plausible manifold of activation-relevant conformations.

Analysis of the resulting ensemble reveals a highly ordered and directional reorganization of the STING dimer interface. Along the pathway, the 2 α1 helices undergo a gradual and coordinated opening motion, with the interhelical distance increasing continuously from ~3.4 nm in the closed state to ~6.0 nm in the open state, and the interhelical angle increasing monotonically from ~49° to nearly 89° (Fig. 2). These geometric changes proceed smoothly across successive intermediates, indicating that the closed–open transition is dominated by collective rotation and separation of preformed α1 helices rather than by abrupt rearrangements or local disorder.

Importantly, the pathway also reveals a staged progression of conformational expansion. Early intermediates (state 1 to state 4) retain a relatively compact architecture with modest changes in α1–α1 geometry, whereas later intermediates (state 5 to state 8) exhibit increasingly pronounced separation and rotation approaching the open configuration. Throughout this progression, secondary structural elements remain largely preserved, indicating that the transition is governed primarily by relative motions among preformed structural units rather than unfolding or refolding events. Together, these features define a connected yet constrained ensemble in which large-amplitude rearrangements occur along preferred collective directions.

Defining this physically accessible ensemble has direct mechanistic implications for how ligand binding can regulate activation. If the activation manifold is already accessible, ligand binding need not “create” new structural states or open a forbidden pathway; instead, it must act on this preexisting substrate to redistribute conformational probabilities and couplings, selectively stabilizing and kinetically privileging particular regions within the ensemble. Establishing the structure of this manifold therefore provides the necessary foundation for examining how C-di-GMP reshapes conformational populations and information flow to bias STING toward activation-competent dynamic regimes.

### Ligand binding suppresses nonproductive fluctuations while preserving large-scale conformational accessibility

Structural accessibility alone does not determine functional activation within the physically accessible conformational ensemble defined above. Rather, activation must emerge from how ligand binding redistributes dynamic weight within this ensemble—selectively suppressing certain motions while preserving others. To resolve this redistribution at the dynamic level, we performed extensive microsecond-scale MD simulations of the STING LBD in both the apo and C-di-GMP-bound states. For each condition, 10 representative starting conformations spanning the closed–open transition were simulated for 1 μs each, yielding an aggregate sampling time of 10 μs per system and enabling ensemble-consistent comparison across identical regions of conformational space.

**Fig. 3.**
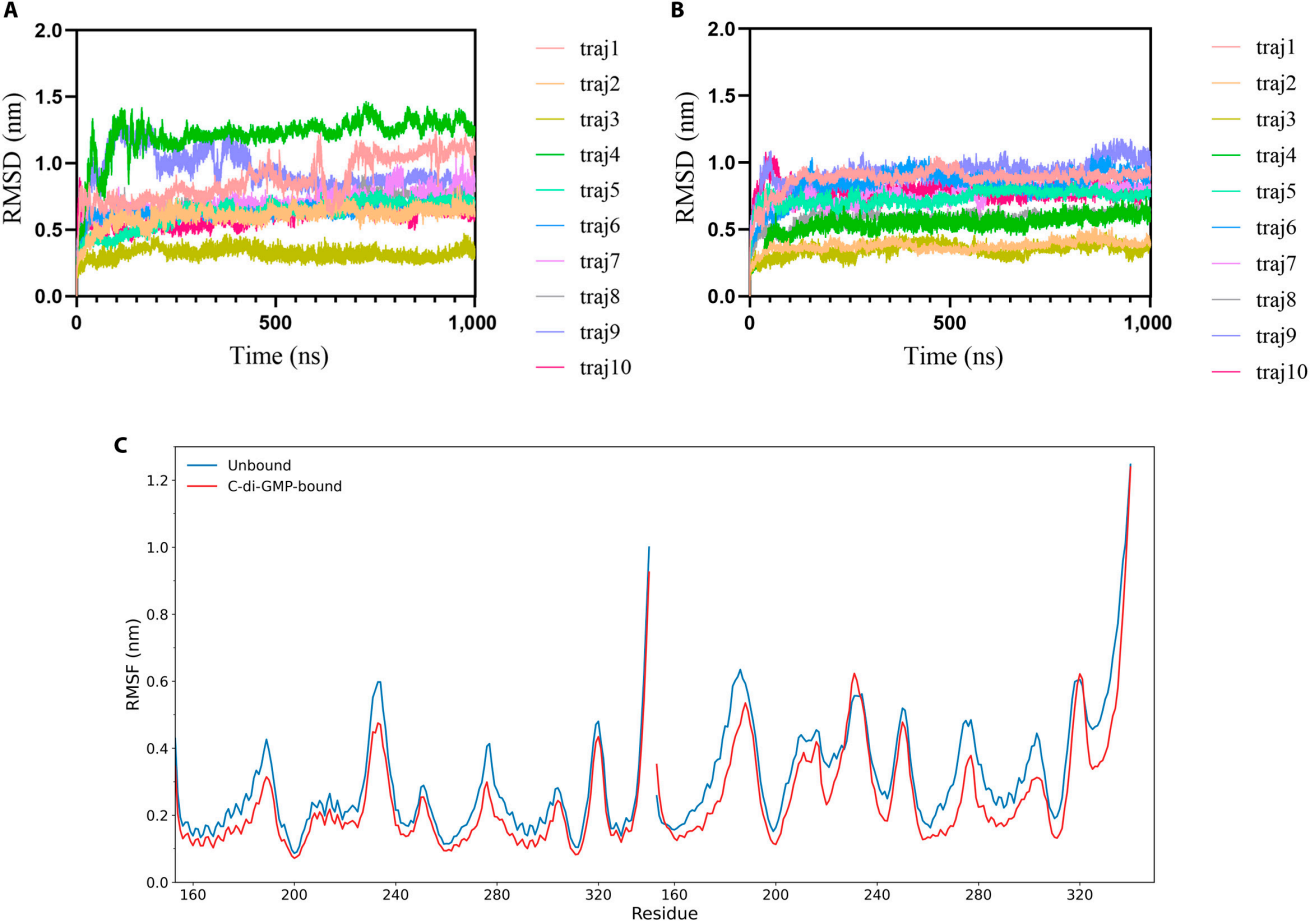
Ligand binding selectively filters conformational fluctuations while preserving activation-relevant dynamics of the STING LBD. (A) Time evolution of backbone root-mean-square deviation (RMSD) for the apo STING ligand-binding domain (LBD) across 10 independent microsecond-scale molecular dynamics trajectories initiated from distinct conformations spanning the closed–open ensemble. Trajectories exhibit pronounced heterogeneity and strong dependence on initial conditions, indicating extensive exploration of conformational space and the presence of large-amplitude, nondirectional fluctuations in the absence of ligand. (B) Backbone RMSD evolution for the C-di-GMP-bound STING LBD under the same multistart simulation protocol. In contrast to the apo system, trajectories rapidly converge to stable RMSD plateaus and display markedly reduced intertrajectory variability. Importantly, this convergence reflects suppression of uncoordinated conformational drift rather than global immobilization, as large-scale rearrangements associated with activation remain accessible. (C) Residue-level root-mean-square fluctuations (RMSF) averaged over all trajectories for the apo (blue) and C-di-GMP-bound (red) systems. Ligand binding leads to a broad attenuation of local, high-frequency fluctuations across the protein, while several regions retain appreciable flexibility. This nonuniform reduction indicates selective damping of nonproductive motions rather than uniform rigidification. Together, these analyses show that C-di-GMP binding reshapes STING LBD dynamics by filtering stochastic and nondirectional fluctuations while preserving collective motions required for activation-relevant conformational transitions.

At the global level, trajectories in the apo system exhibit pronounced heterogeneity and strong dependence on the initial conformation. Although most trajectories rapidly depart from their starting structures, they fail to converge to a common dynamic regime on the microsecond timescale (Fig. 3A). Instead, individual trajectories stabilize at distinct RMSD plateaus, spanning a broad range from relatively compact configurations to highly expanded geometries. This dispersion indicates that, in the absence of ligand, STING LBD explores a wide spectrum of conformations within the accessible ensemble, including large-amplitude fluctuations that are weakly constrained and not directionally aligned with productive activation.

In contrast, trajectories in the C-di-GMP-bound system display a markedly different dynamic signature. Independent simulations initiated from diverse starting conformations rapidly converge to more similar RMSD plateaus and maintain more consistent dynamic behavior throughout the simulations (Fig. 3B). Importantly, this convergence does not reflect global immobilization of the protein. Instead, it reflects suppression of uncoordinated, nondirectional conformational drift. The ligand-bound system retains access to large-scale rearrangements associated with α1-helix reorientation, while exhibiting substantially reduced intertrajectory variability and long-term fluctuations.

Because the RMSD profiles in Fig. 3A and B were calculated relative to the initial structure of each corresponding trajectory, they quantify how far each simulation departs from its own starting conformation rather than directly measuring similarity to the experimentally resolved closed or open endpoint structures. To place these fluctuations in the context of the canonical STING LBD endpoints, we additionally calculated backbone RMSDs relative to the processed closed and open crystal structures, corresponding to PDB 4F5D and PDB 4F5Y, respectively (Fig. S6). These endpoint-referenced RMSD analyses provide a complementary frame of reference for evaluating whether simulated conformations are closer to closed-like, open-like, or intermediate geometries. Together with the initial-structure-referenced RMSD profiles, these comparisons indicate that the simulations should be interpreted as ensemble-level conformational exploration across the closed–open manifold rather than as relaxation around a single endpoint structure.

As an additional control for the ligand-bound simulations, we monitored the positional stability of C-di-GMP by calculating ligand heavy-atom RMSD in 3 representative STING–C-di-GMP complex trajectories initiated from the closed conformation, one intermediate conformation, and the open conformation (Fig. S7). For each trajectory, the ligand RMSD was calculated after alignment to the protein backbone, so that the resulting values reflect the stability of C-di-GMP relative to the STING LBD binding pocket. C-di-GMP remained stably bound in the binding site in all 3 representative conformational contexts, and no positional restraints were applied to the ligand during production MD. These results support that the docked C-di-GMP pose is stable in representative closed, intermediate, and open-like STING LBD environments, indicating that the ligand-induced changes in protein dynamics are not attributable to ligand dissociation or an unstable binding pose.

To further connect the global RMSD changes to the activation-related geometry defined in Fig. 2, we monitored the α1-α1 separation distance and the interhelical angle as functions of simulation time across all 10 independent trajectories in both the C-di-GMP-bound and unbound systems (Fig. S8). This analysis was performed at the ensemble level rather than using a single representative trajectory. The time-series profiles showed that both descriptors sampled broad conformational ranges across independent simulations. In both systems, different trajectories occupied distinct regions of the α1-mediated conformational space, indicating that the observed α1-helix separation and rotation were not restricted to an individual trajectory.

To further summarize the trajectory-level sampling behavior, we analyzed the distributions of the α1–α1 distance and interhelical angle for each independent trajectory using boxplots (Fig. S9). These distribution analyses confirmed that the conformational heterogeneity observed in the time-series profiles was reproducibly captured across the simulation ensemble. Individual trajectories sampled different portions of the conformational space, collectively covering compact, intermediate, and more expanded α1 arrangements. This result supports the presence of multiple α1-helix geometries rather than fluctuations around a single narrowly defined conformation.

Finally, we reconstructed 2-dimensional free-energy landscapes using the pooled trajectories from each system, with the α1–α1 distance and interhelical angle as reaction coordinates (Fig. S10). The pooled free-energy landscapes revealed multiple low-free-energy basins corresponding to structurally distinct α1 arrangements in both the C-di-GMP-bound and unbound systems. Together, these ensemble-level analyses indicate that the structurally distinct α1-helix geometries discussed in the manuscript are supported by the full set of independent simulations and are not artifacts of one selected trajectory.

Residue-level analysis further supports this selective redistribution of dynamics. In the apo state, elevated RMSF values are observed across multiple regions, particularly in loop segments and secondary-structure junctions, reflecting widespread local flexibility (Fig. 3C). Upon C-di-GMP binding, RMSF values are broadly reduced, indicating attenuation of local, high-frequency fluctuations. Notably, this reduction is not uniform: several regions retain appreciable flexibility, consistent with preservation of the collective motions required for large-scale conformational rearrangement. Together, these observations indicate that ligand binding preferentially dampens nonproductive local fluctuations while maintaining the structural plasticity necessary for activation-relevant motions.

Taken together, the protein RMSD, endpoint-referenced RMSD, ligand RMSD, α1-helix geometric descriptors, and RMSF analyses demonstrate that C-di-GMP binding reshapes the dynamical landscape of the STING LBD not by globally rigidifying the protein, but by selectively redistributing dynamic weight within a preexisting, physically accessible conformational ensemble. The stability of C-di-GMP in representative ligand-bound trajectories further supports that these dynamic effects arise from a persistent ligand-bound state rather than from ligand dissociation. While fluctuation-based measures reveal how ligand binding modulates the amplitude and coherence of motion, they do not resolve how specific conformations become preferentially populated or how transitions between them are reorganized. Resolving these state-level effects is therefore essential for understanding how dynamic redistribution is converted into functional activation.

### Energy landscape reweighting biases STING toward intermediate, activation-competent dynamic regimes

**Fig. 4.**
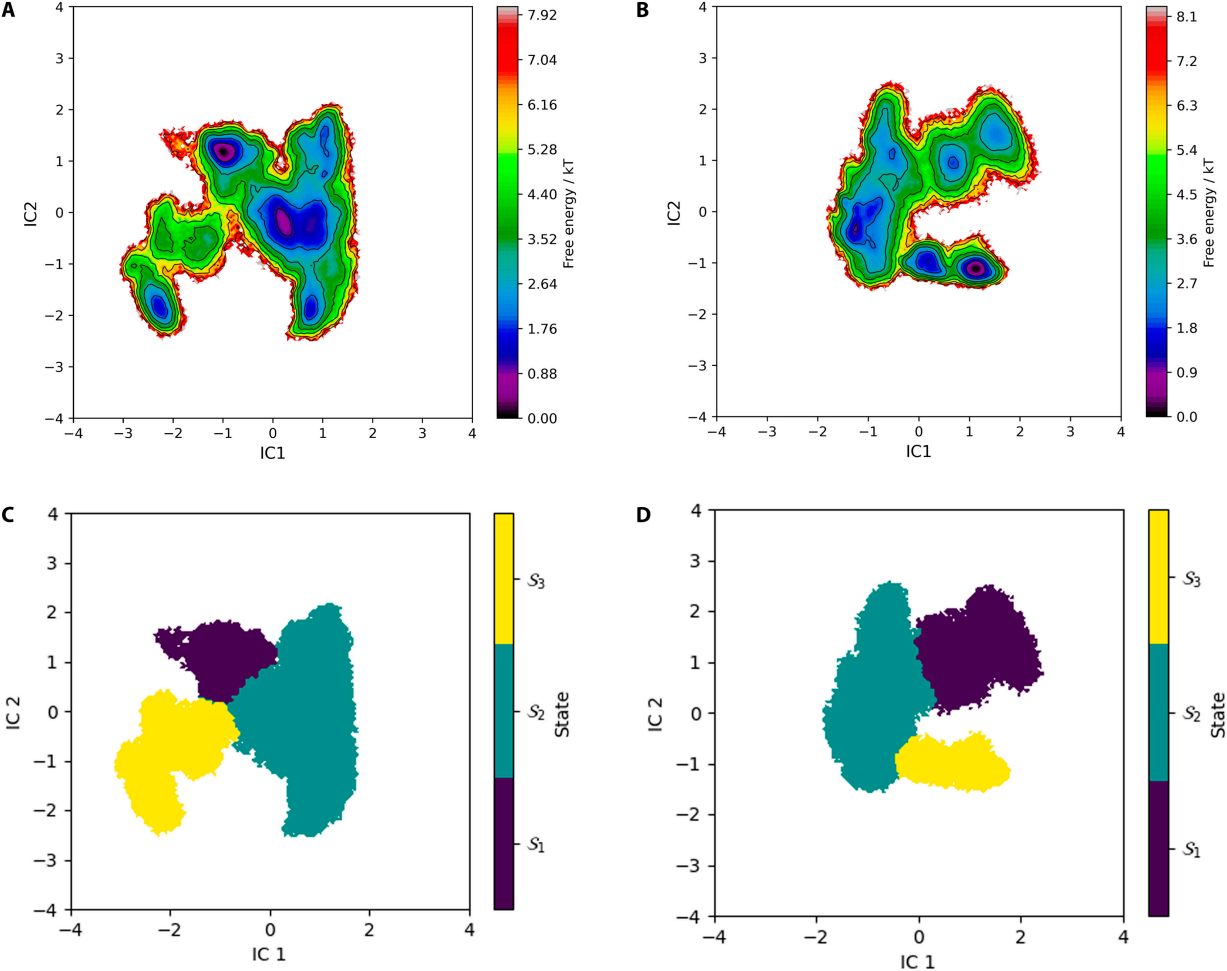
Ligand binding reweights the conformational free-energy landscape and stabilizes activation-relevant macrostates of the STING LBD. (A and B) Conformational free-energy landscapes reconstructed from Markov state models (MSMs) and projected onto the first 2 time-lagged independent components (IC1 and IC2) for the apo STING ligand-binding domain (LBD) (A) and the C-di-GMP-bound system (B). Colors indicate relative free energy in units of kT, with low-free-energy regions corresponding to thermodynamically favored conformational basins. Contour lines were drawn at 1-kT intervals starting from 1 kT to improve visualization of basin boundaries and free-energy gradients. In the apo system, the landscape is broad and fragmented, featuring multiple low-energy basins distributed across an extended region of conformational space. Upon C-di-GMP binding, the accessible landscape becomes more compact, with low-energy regions concentrated into a smaller subset of IC space. (C and D) Macrostate decomposition of the MSMs using PCCA+ clustering for the apo (C) and C-di-GMP-bound (D) systems. Three dominant macrostates (S1 to S3) are identified in each case and mapped onto IC space. In the apo system, macrostates occupy distinct and widely separated regions, reflecting a heterogeneous ensemble with substantial accessibility of expanded conformational basins. In contrast, ligand binding is associated with a redistribution of macrostate organization, with population shifted toward more compact and intermediate conformational regimes and reduced occupation of highly expanded regions. Together, these results support that C-di-GMP binding is associated with thermodynamic reweighting of the STING LBD conformational landscape, favoring a more restricted subset of conformational states. This ligand-associated redistribution of conformational populations provides a quantitative ensemble-level context for the altered kinetic connectivity and dynamic coupling analyses described below.

**Fig. 5.**
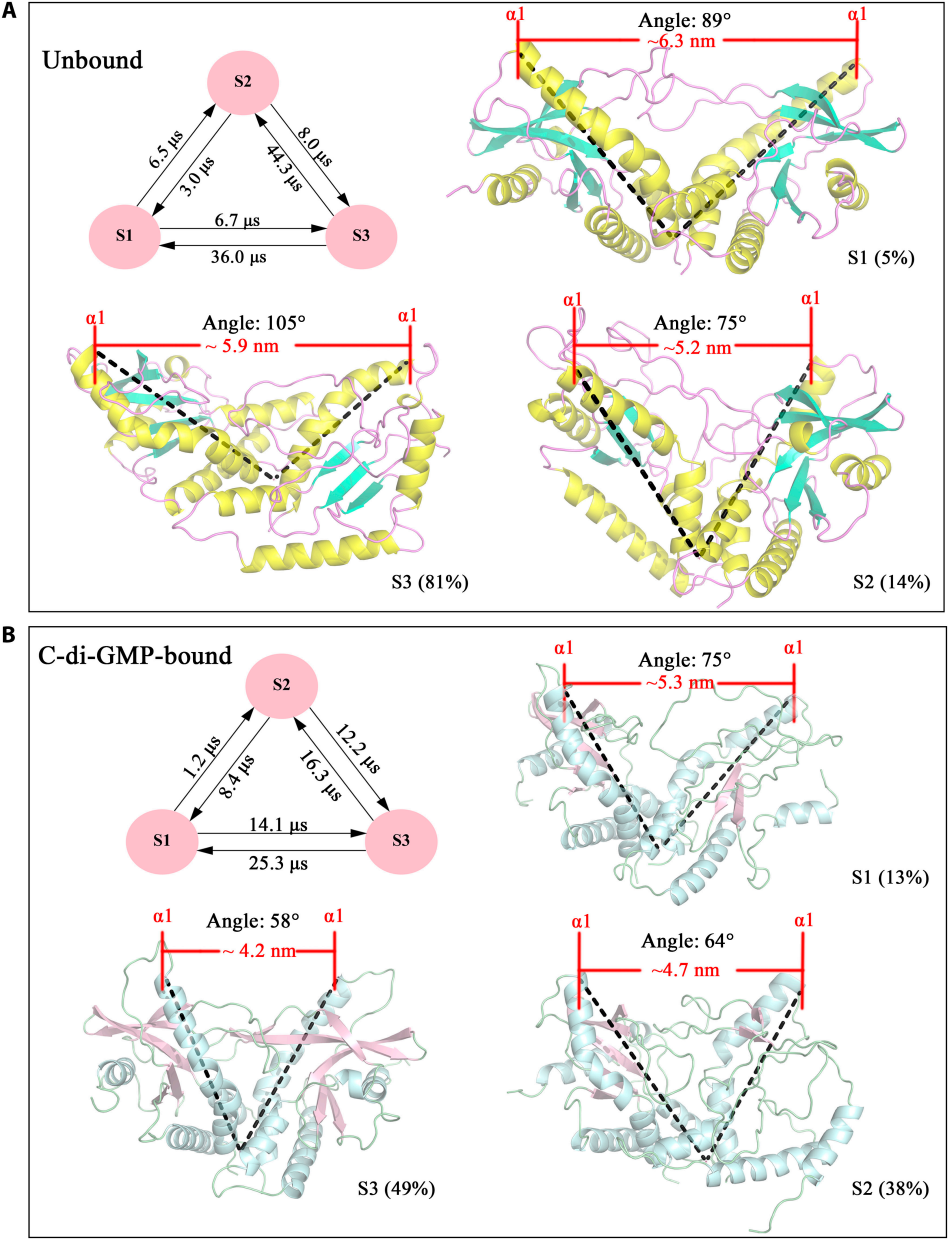
Ligand-induced reorganization of macrostate populations and kinetic connectivity in the STING LBD conformational ensemble. (A) Macrostate transition network of the apo STING ligand-binding domain (LBD) reconstructed from the Markov state model (MSM), together with representative conformations of each macrostate. Circles denote macrostates (S1 to S3), arrows indicate dominant interstate transitions, and labels report the mean transition timescales (μs). Representative structures of each macrostate are shown on the right, with their equilibrium populations indicated. In the apo ensemble, conformational populations are highly uneven, with the majority of the ensemble residing in an expanded macrostate (S3), while transitions between states occur through multiple pathways with disparate timescales. (B) Corresponding macrostate transition network and representative conformations for the C-di-GMP-bound STING LBD. Ligand binding markedly redistributes macrostate populations, reducing dominance of the most expanded basin and stabilizing intermediate conformations with geometries closer to activation-relevant arrangements. At the same time, the transition network becomes kinetically more focused, with reduced path redundancy and more balanced interconversion timescales between macrostates. In the representative structures, red annotations indicate the angle (°) and distance (nm) between the 2 α1 helices, and black dashed lines highlight their relative orientations. Comparison between the apo and ligand-bound systems reveals that C-di-GMP binding does not simply restrict conformational motion, but selectively biases both the thermodynamic populations and kinetic accessibility of macrostates toward conformations compatible with productive activation. This kinetic reorganization provides a direct bridge between ensemble reweighting and subsequent remodeling of allosteric communication pathways.

If ligand-induced dynamic redistribution is associated with functional activation, it should be reflected in how conformational states are differentially populated and how transitions between them are organized. Fluctuation-based analyses alone cannot resolve these questions, as they do not distinguish between transient stabilization and genuine reweighting of the conformational ensemble. To capture these state-level effects, a framework is required that explicitly resolves both the thermodynamic organization of conformational states and the kinetics of their interconversion across the activation-relevant ensemble. To this end, we constructed MSMs from the multistart, microsecond-scale MD trajectories and analyzed the resulting conformational landscapes and transition kinetics in the space of time-lagged ICs (Figs. 4 and 5).

Before interpreting the MSM-derived thermodynamic and kinetic properties, we first evaluated the robustness of the MSM construction. Transition probability matrices were estimated at a lag time of 5.5 ns. ITS analysis showed that the dominant slow processes reached an approximately stable regime beyond ~5 ns, supporting the use of 5.5 ns as the MSM lag time (Fig. S2). The ITS profiles were computed with Bayesian uncertainty estimates, and the corresponding confidence intervals indicate that the leading slow processes are separated from the selected lag time. Chapman–Kolmogorov validation further showed good agreement between MSM-predicted and directly estimated transition probabilities among the 3 macrostates, supporting the Markovian behavior of the coarse-grained models at the selected lag time (Fig. S3).

To quantify statistical uncertainty in the MSM-derived quantities, we estimated posterior distributions of macrostate populations and MFPTs using Bayesian MSM sampling. The posterior distributions of macrostate populations were narrow and well separated for both the apo and C-di-GMP-bound systems (Fig. S5), supporting the robustness of the inferred equilibrium reweighting. The MFPT posterior distributions were unimodal, with the apo system showing transitions on the order of ~30 to 40 μs and the C-di-GMP-bound system showing transitions on the order of ~20 to 30 μs (Fig. S4). Although these MSM-inferred transition times exceed the length of individual trajectories, they are estimated by integrating transition statistics across all independent trajectories under the validated Markov model. Therefore, the transition times reported below should be interpreted as MSM-inferred kinetic estimates with posterior uncertainty, rather than as directly observed transition events within a single trajectory.

For mechanistic interpretation, the MSM microstates were coarse-grained into 3 macrostates using PCCA+. This 3-macrostate representation was used because it provided a physically interpretable coarse-graining of the dominant conformational basins sampled in the TICA/MSM landscape, corresponding to compact, intermediate, and expanded/open-like conformational regimes. We therefore use the 3-state model as a mechanistically interpretable representation of the sampled conformational landscape, rather than as a uniquely determined state decomposition.

Because the primary MSM was constructed using β2–β2 Cα distance features, we further assessed whether the main conclusions depend on this specific feature representation. To this end, we constructed an additional MSM using interchain interface contact features, which provide a broader description of dimer-interface rearrangements. Interface contacts between chains A and B were encoded using 2 distance thresholds, 0.4 and 0.7 nm, to capture both close and intermediate interfacial interactions. The same TICA projection, microstate clustering, lag-time validation, and PCCA+ coarse-graining workflow were then applied to this alternative feature set. Importantly, the interface-contact-based MSM reproduced the same qualitative ligand-dependent conformational reweighting observed in the primary β2-distance-based MSM. Specifically, the apo ensemble remained enriched in more expanded/open-like conformational regions, whereas the C-di-GMP-bound ensemble was shifted toward more compact and intermediate conformational regimes with reduced occupation of highly expanded states (Fig. S11). These results support that the central conclusion of ligand-induced ensemble reweighting is robust to feature choice and is not an artifact of the β2–β2 distance representation.

Having evaluated the MSM construction, uncertainty estimates, and feature robustness, we next examined the ligand-dependent conformational landscapes. Projection of the apo STING LBD ensemble onto the first 2 ICs reveals a highly dispersed free-energy landscape characterized by multiple, well-separated low-free-energy basins (Fig. 4A). These basins correspond to distinct metastable conformational states and collectively span a broad region of conformational space, indicating that, in the absence of ligand, the STING LBD populates a heterogeneous ensemble with no single dominant energetic basin. Coarse-graining of MSM microstates using PCCA+ identifies 3 macrostates (S1 to S3) (Fig. 4C), with the conformational population strongly biased toward highly expanded geometries. In particular, the S3 state dominates the apo ensemble, with a posterior population of approximately 81%, whereas S2 and S1 account for approximately 14% and 5%, respectively; the corresponding 95% credible intervals are provided in Fig. S5.

Structural characterization of these macrostates shows that this population bias reflects preferential stabilization of expanded conformations. The dominant S3 macrostate corresponds to a highly expanded geometry, with an α1–α1 helix angle of ~105° and an interhelical separation of ~5.9 nm, whereas S2 represents a moderately expanded conformation (angle ~75°, distance ~5.2 nm) and S1 corresponds to an even more expanded/open configuration (angle ~89°, distance ~6.3 nm) (Fig. 5A). Bayesian MSM analysis further indicates that interconversion among these macrostates occurs on the order of tens of microseconds, with posterior MFPT distributions summarized in Fig. S4. In the apo system, transitions are relatively slow and broadly distributed, and S2 serves as a kinetically connected intermediate between expanded conformational basins. Together, these features indicate that the apo STING LBD explores an energy landscape that is both thermodynamically dispersed and kinetically permissive, favoring extensive sampling of highly expanded conformations.

In contrast, ligand binding is associated with a pronounced reorganization of this landscape. In the C-di-GMP-bound system, the free-energy surface becomes markedly more compact, with low-free-energy regions concentrated within a narrower region of IC space (Fig. 4B). Although 3 macrostates are again identified by PCCA+ clustering (Fig. 4D), their populations are fundamentally redistributed. In the ligand-bound ensemble, S3 and S2 account for approximately 49% and 38% of the population, respectively, whereas the most expanded S1 state is reduced to approximately 13%; the corresponding 95% credible intervals are provided in Fig. S5.

Representative structures reveal that ligand binding is associated with reduced occupation of extremely expanded geometries and increased stabilization of compact and intermediate conformations. In the bound system, the dominant S3 macrostate adopts a relatively compact configuration (α1–α1 angle ~58°, distance ~4.2 nm), while S2 corresponds to a moderately expanded conformation (angle ~64°, distance ~4.7 nm). The residual S1 population corresponds to a more expanded state (angle ~75°, distance ~5.3 nm), but this geometry is no longer the dominant energetic basin (Fig. 5B). Thus, rather than enforcing a single rigid closed structure, C-di-GMP binding reshapes the energy landscape to concentrate population within a restricted band of compact-to-intermediate conformations that lie between the closed and fully open extremes.

Reorganization of conformational populations is accompanied by a concomitant restructuring of transition kinetics. In the C-di-GMP-bound system, MSM-inferred MFPTs are overall shorter and the transition architecture becomes more focused, with dominant kinetic routes passing through a restricted subset of macrostates (Fig. 5B). Notably, the S2 macrostate emerges as both highly populated and kinetically central, exhibiting relatively short MFPTs to neighboring states within the Bayesian posterior ensemble. This behavior contrasts with the apo system, in which transitions are slower and more distributed across macrostates. The emergence of a kinetically privileged intermediate suggests that ligand binding not only biases state populations, but also reorganizes how conformational transitions are routed through the ensemble.

Taken together, the Bayesian-supported MSM analysis indicates a coordinated reshaping of both thermodynamic and kinetic organization during STING activation. In the absence of ligand, the conformational landscape is dispersed and biased toward highly expanded conformations, with slower and more distributed MSM-inferred transitions. Upon C-di-GMP binding, this landscape is selectively reweighted to reduce highly expanded conformations, stabilize compact-to-intermediate states, and focus transitions through a kinetically privileged region of conformational space. The ITS convergence, CK validation, Bayesian posterior uncertainty analyses, and alternative interface-contact-based MSM collectively support that these population shifts and MFPT estimates are robust within the sampled ensemble and are not artifacts of the specific β2–β2 distance feature representation. This state-resolved reorganization provides quantitative support for a model in which ligand-induced dynamic redistribution is associated with a biased ensemble architecture that supports productive activation.

### Ligand-induced activation is accompanied by reorganization of long-range allosteric communication networks

The ensemble-level reweighting of conformational states and transition kinetics described above is expected to be accompanied by changes in the underlying residue–residue interaction architecture. In protein systems, thermodynamic bias and kinetic focusing are closely related to how local interactions are structured, coupled, and coordinated across the molecular scaffold. We therefore asked whether ligand binding restructures the internal interaction network of the STING LBD in a manner associated with the observed redistribution of conformational populations and transition pathways.

**Fig. 6.**
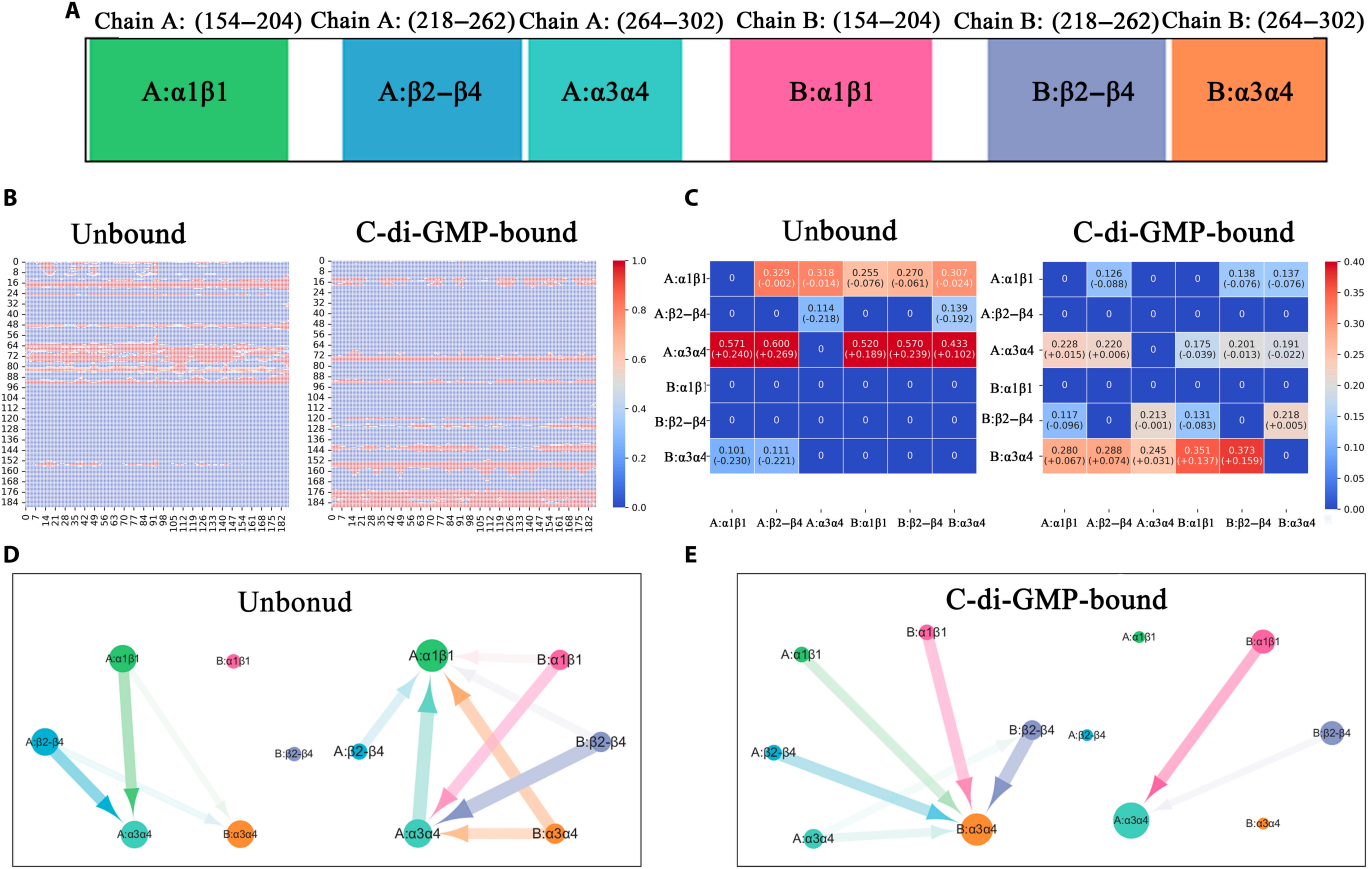
Ligand-induced reorganization of neural relational inference (NRI)-inferred dynamic coupling architecture within the STING LBD. (A) Definition of 6 functional regions used for region-level network analysis of the STING LBD dimer. The LBD was divided according to secondary-structure organization and chain identity into A:α1β1, A:β2–β4, A:α3α4, B:α1β1, B:β2–β4, and B:α3α4. These regions were used to coarse-grain residue-level NRI-inferred couplings into region-averaged coupling matrices and directed regional networks. (B) Residue-level dynamic coupling maps inferred by NRI for the unbound and C-di-GMP-bound STING LBD systems. The blue-to-red color scale represents inferred coupling strength or edge probability between residue pairs, with blue indicating weak or absent coupling and red indicating stronger dynamical coupling. Compared with the unbound system, C-di-GMP binding redistributes residue-level coupling patterns across the LBD dimer. (C) Region-averaged NRI coupling matrices for the unbound and C-di-GMP-bound systems. The 6 functional regions defined in panel (A) are shown on both axes. Matrix colors indicate the averaged coupling strength between pairs of regions, using a blue-to-red scale from weak to strong coupling. Cell values report the corresponding region-averaged coupling strengths. The comparison shows that C-di-GMP binding does not uniformly increase all couplings, but instead reorganizes coupling strengths among specific functional regions.(D and E) Directed region-level NRI coupling networks for the unbound (D) and C-di-GMP-bound (E) systems, constructed from the region-averaged coupling matrices. Nodes represent the 6 functional regions, with node colors matching the region definitions in panel (A). Arrows indicate directed dynamical couplings inferred by NRI, and arrow thickness reflects the relative coupling strength. In the unbound system, couplings are more broadly distributed and relatively diffuse, whereas in the C-di-GMP-bound system, the coupling architecture is reorganized into a more selective pattern of interregional communication.

To address this question, we inferred dynamic residue–residue interaction networks directly from MD trajectories using an NRI framework. This data-driven approach reconstructs effective dynamic couplings without presupposing static contacts or predefined signal routes, thereby capturing how interaction architectures adapt in response to ligand binding. Importantly, this NRI analysis was performed independently of the MSM state decomposition described above. Whereas MSM analysis was used to characterize metastable conformational states, equilibrium populations, and transition kinetics, NRI was used to infer ensemble-level dynamic coupling architectures directly from trajectory windows. The NRI model was trained using all 10 independent 1-μs trajectories for each condition, and the networks shown in Fig. 6 therefore represent ensemble-level apo and C-di-GMP-bound interaction architectures rather than MSM macrostate-specific or single-trajectory networks.

To facilitate mechanistic interpretation, the STING LBD was partitioned into 6 functional modules corresponding to major secondary-structure elements across the dimer: the α1β1, β2–β4, and α3α4 regions of each monomer (Fig. 6A). This region-resolved representation allows network reorganization to be interpreted in terms of coordinated coupling between structurally and functionally distinct elements, rather than as isolated residue-level effects.

In the apo state, the inferred interaction network exhibits a diffuse and weakly organized topology (Fig. 6B and D). Coupling strengths between functional modules are broadly distributed, with no single region or interface emerging as a dominant interaction hub. Although interchain interactions are present, they remain relatively fragmented and weak, indicating that long-range coupling is insufficiently organized to impose strong directional constraints on conformational dynamics. This interaction architecture is consistent with the thermodynamically dispersed and kinetically permissive ensemble observed in the absence of ligand binding.

Upon C-di-GMP binding, the interaction network undergoes a pronounced and selective reorganization (Fig. 6C and E). Rather than uniformly strengthening all contacts, ligand binding redistributes coupling strength toward specific interregional connections, giving rise to a more structured and hierarchical network topology. In particular, couplings involving the β2–β4 and α3α4 regions are selectively reorganized, while interactions involving the α1β1 modules become concentrated into fewer and more coherent channels. This pattern indicates that ligand binding reshapes the interaction architecture to favor preferential routes of dynamic coupling, rather than simply increasing overall network connectivity.

Importantly, this reorganization extends beyond the immediate vicinity of the ligand-binding site. Reinforced couplings propagate across the dimer and alter the coordination between spatially distant but dynamically connected regions. By reducing the apparent dimensionality of the coupling landscape, C-di-GMP binding transforms a diffuse interaction network into one dominated by a more restricted set of coordinated interregional connections. Such an architecture is consistent with a dynamic substrate that may contribute to the stabilization of compact-to-intermediate conformational regimes and to the organization of conformational transitions along preferred collective directions, as indicated by the MSM analysis.

To evaluate whether the NRI-derived network reorganization depended on the edge-probability threshold used to define marked couplings, we repeated the analysis using a relaxed threshold of 0.1, compared with the 0.2 threshold used in the primary analysis. Under this more permissive cutoff, the major qualitative differences between the apo and C-di-GMP-bound systems were preserved (Fig. S12). The apo network retained a relatively diffuse coupling architecture, whereas the C-di-GMP-bound network continued to show a reorganized pattern of interregional couplings. The preservation of these apo-versus-bound differences at a lower edge-probability threshold indicates that the inferred ligand-induced network remodeling is not an artifact of the specific 0.2 cutoff used in the primary analysis.

We further compared the NRI-derived coupling architecture with DCCM analysis, a conventional method for characterizing pairwise linear correlations in atomic fluctuations. The DCCM heatmaps revealed extensive correlated and anti-correlated motions in both apo and C-di-GMP-bound systems, and the corresponding region-level DCCM networks remained relatively dense in both cases (Fig. S13). Thus, DCCM provides a useful reference for global fluctuation correlations, but its dense regional networks make it difficult to resolve selective information-transfer routes or pathway-level focusing. In contrast, NRI infers predictive dynamic dependencies from trajectory windows and yields a more structured coupling architecture suitable for downstream shortest-path and information-flow analysis. This comparison highlights the added value of the NRI framework for identifying selective, pathway-resolvable allosteric communication beyond broad pairwise correlation patterns.

Taken together, the NRI network analysis indicates that ligand binding restructures the internal interaction topology of the STING LBD from a weakly organized and permissive network into a more coherent and hierarchically coupled architecture. The threshold-sensitivity analysis supports that this network reorganization is robust to a relaxed edge-probability cutoff, while the DCCM comparison demonstrates that conventional correlation analysis captures broad dynamic correlations but does not readily resolve the sparse, pathway-level communication architecture inferred by NRI. By redefining how structural modules are dynamically coupled across the protein, C-di-GMP binding establishes an interaction framework that can support more efficient long-range information transfer, whose geometric routing and path-level focusing are examined in the following section.

### Network reorganization focuses allosteric information flow to enable productive activation

While ligand binding reorganizes the interaction architecture of the STING LBD, functional activation ultimately depends on whether this architecture enforces directional and reliable allosteric communication. Network remodeling becomes mechanistically consequential only if it collapses the degeneracy of communication routes, such that signals originating near the ligand-binding site are preferentially transmitted to distal structural elements that govern dimer opening. We therefore examined how C-di-GMP-induced network reorganization reshapes the dominant communication pathways linking the β2–β4 region to the mechanically responsive α1β1 modules.

To this end, we analyzed shortest allosteric communication paths extracted from the NRI-inferred interaction networks. Edge weights were defined as the inverse of interaction strength, such that shortest paths correspond to routes of maximal effective coupling. Communication was traced from the β2–β4 region of chain A, which lies proximal to the ligand-binding site, to the α1β1 modules of both monomers, which act as the primary mechanical elements controlling dimer opening.

**Fig. 7.**
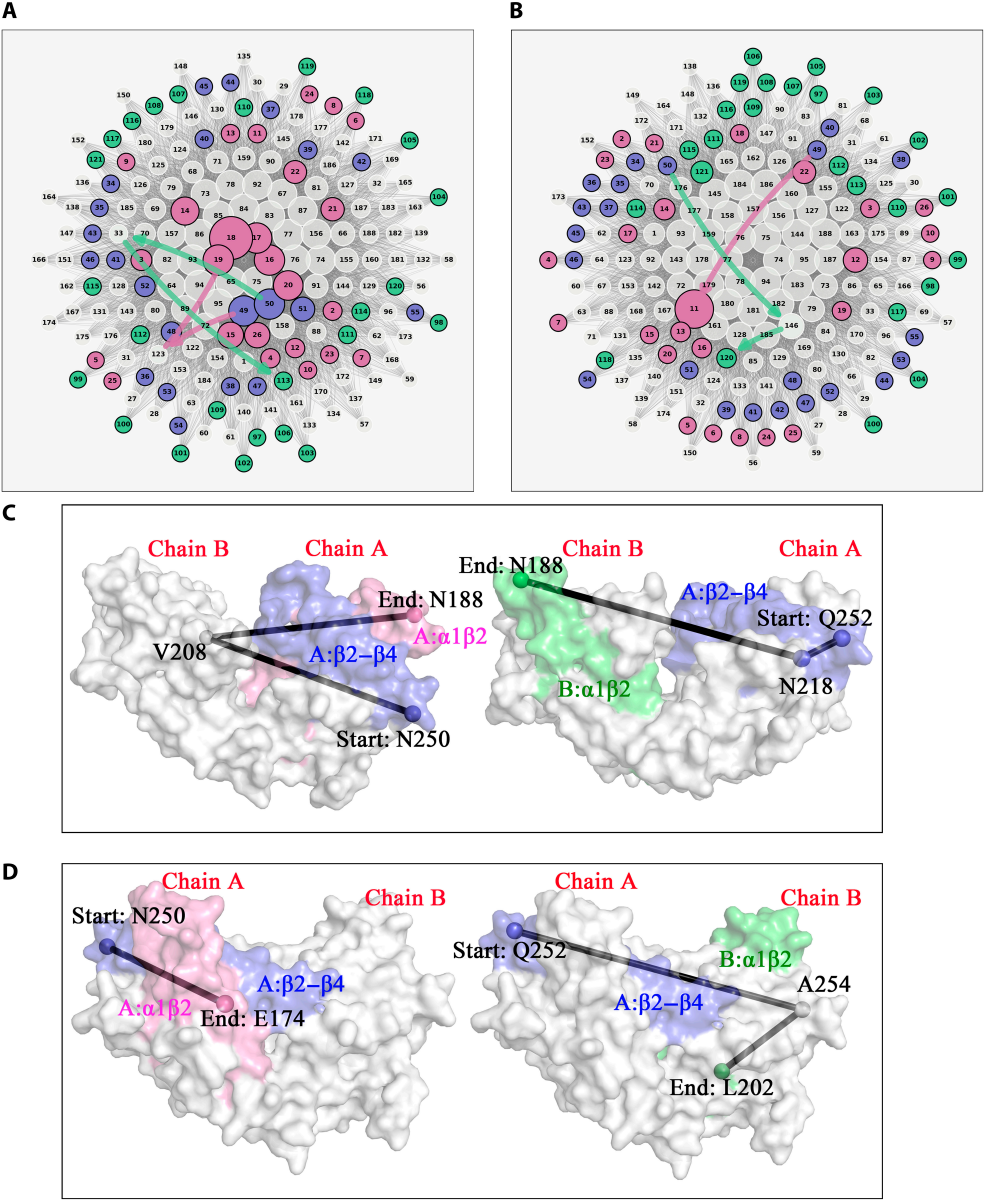
Ligand-induced focusing and shortening of allosteric information pathways in the STING LBD. (A) Concentric network representation of shortest allosteric communication pathways connecting the A-chain β2–β4 region to the α1β1 regions of chains A and B in the apo STING ligand-binding domain (LBD). Nodes are arranged radially according to centrality, reflecting their relative importance in signal propagation. Directed paths were computed using edge-weighted shortest-path analysis, with edge weights defined as the inverse of dynamically inferred interaction strength. (B) Concentric network representation of shortest communication pathways in the C-di-GMP-bound STING LBD. Upon ligand binding, the dominant communication routes become shorter and more direct, indicating a reorganization toward more efficient signal propagation. (C) Spatial mapping of representative shortest paths from the apo system onto the STING LBD molecular surface. In the absence of ligand, communication from the A-chain β2–β4 region to the α1β1 modules relies on cross-chain, interface-mediated detour pathways, reflecting relatively diffuse and indirect information transmission. (D) Spatial mapping of representative shortest paths in the C-di-GMP-bound system. Ligand binding collapses these detour-like routes into more compact pathways that are predominantly intrachain or involve intrachain intermediates on the target chain, thereby reducing path length and minimizing reliance on cross-chain bridging. Together, these analyses demonstrate that C-di-GMP binding does not merely alter interaction strengths, but fundamentally reorganizes how allosteric information is routed through the STING LBD. By shortening communication paths and shifting propagation from interface-dependent detours to focused intrachain routes, ligand binding enhances the efficiency and reliability of long-range signal transmission, providing a direct mechanistic link between information flow reprogramming and conformational activation.

In the apo state, allosteric communication exhibits a highly degenerate and circuitous architecture (Fig. 7A and C). Signal propagation from the ligand-proximal region to the α1β1 modules is distributed across many alternative routes, frequently involving cross-chain detours through interface residues. Quantitatively, the communication probability is broadly spread across numerous low-weight paths, with no single route dominating information transfer (Table 1). These paths are relatively long, traverse multiple intermediate nodes, and each carries only a small fraction of the total communication probability. Such redundancy indicates that long-range communication is weakly constrained and inefficient, consistent with the thermodynamically dispersed ensemble and unfocused transition kinetics observed in the absence of ligand.

**Table 1.** Dispersed and redundant allosteric communication pathways in apo STING LBD

A:β2–β4−A:α1β1		A:β2–β4−B:α1β1	
Pathways (resi)	Probability	Pathways (resi)	Probability
250-208-188	0.2069	252-218-188	0.2932
252-208-188	0.1977	252-256-184	0.2892
250-208-184	0.1642	254-294-184	0.1661
252-208-184	0.1570	254-294-188	0.1418
254-208-188	0.0960	250-256-184	0.0959
254-328-190	0.0811	254-330-204-186	0.0092
252-208-184	0.0762	250-296-160-218-188	0.0024
250-330-328-190	0.0113	250-246-336-204-186	0.0013
252-326-328-190	0.0096	252-326-328-204-186	0.0011

C-di-GMP binding is associated with a pronounced redistribution of communication routes, consistent with reduced pathway degeneracy. In the ligand-bound state, shortest paths become markedly shorter and more concentrated, with a small number of dominant routes emerging that rely primarily on intrachain connections or locally mediated steps within the target chain (Fig. 7B and D). This collapse is accompanied by a pronounced redistribution of communication probability, such that 1 or 2 pathways account for the majority of signal transmission (Table 2). Cross-chain detours that dominate the apo network are largely eliminated, and information flow is redirected along compact, structurally coherent pathways. This reorganization reflects a transition from permissive, interface-dependent communication to a constrained architecture that enforces directed signal propagation.

**Table 2.** Focused and dominant allosteric communication pathways upon C-di-GMP binding

A:β2–β4−A:α1β1		A:β2–β4−B:α1β1	
Pathways (resi)	Probability	Pathways (resi)	Probability
250-174	0.4738	252-254-202	0.5742
252-288-176	0.3363	250-208-254-202	0.1645
252-218-196	0.1336	250-216-204	0.1643
250-208-218-196	0.0174	250-216-336-188	0.0553
254-254-248-174	0.0168	254-292-194-202	0.0307
252-254-248-174	0.0151	252-154-334-216-204	0.0064
250-214-328-288-176	0.0034	254-154-334-216-204	0.0038
254-292-236-248-176	0.0027	252-254-256-246-289-188	0.0007
254-292-194-218-176	0.0009	254-292-256-322-264-330-289-188	2.1804 - 06

Importantly, this effect is not merely topological but probabilistic. Comparison of the apo and ligand-bound systems reveals a transition from a diffuse probability distribution over many competing routes (Table 1) to a focused distribution dominated by a small subset of high-fidelity pathways (Table 2). As a result, information flow in the ligand-bound network becomes both more efficient and more reliable, reducing signal dissipation and minimizing uncertainty in long-range coupling. Path focusing thus transforms a permissive communication landscape into a functionally committed signaling architecture.

Notably, residues constituting these dominant communication routes overlap with regions that become thermodynamically stabilized and kinetically privileged upon ligand binding. In this way, network reorganization, ensemble reweighting, and pathway focusing converge on the same intermediate conformational regimes identified by MSM analysis. Rather than acting independently, these layers collectively constrain how structural fluctuations are coordinated, ensuring that remaining motions are preferentially aligned with activation-relevant directions.

Taken together, shortest-path analysis indicates that C-di-GMP binding is associated with reduced communication-route degeneracy and more focused information transfer within the NRI-inferred dynamic coupling network. These results provide a pathway-level interpretation of how ligand-dependent network reorganization may be linked to the biased conformational dynamics observed in the MSM analysis. However, because the present analysis does not directly perturb individual residues or inferred coupling edges, these pathways should be interpreted as model-derived communication routes that support, rather than prove, a causal mechanism of activation.

## Discussion

Allosteric regulation has traditionally been interpreted through the lens of discrete structural transitions [55], in which ligand binding stabilizes a predefined active conformation relative to an inactive state. While this framework has been instrumental in organizing structural observations, it offers limited insight into how activation emerges from the intrinsically dynamic and heterogeneous nature of protein ensembles. Here, we demonstrate that STING activation cannot be adequately described as a binary conformational switch [15]. Instead, activation emerges from ligand-induced reprogramming of interaction architecture that focuses internal information flow, thereby reshaping conformational energetics and kinetics within a physically accessible ensemble.

Our analyses reveal that the closed and open conformations of the STING LBD are not separated by an abrupt structural barrier, but are connected by a continuous manifold of geometrically and energetically accessible conformations. This continuity defines the structural substrate on which allosteric regulation must operate, but does not by itself determine activation. Rather than requiring the creation of a new structural state, activation proceeds through selective stabilization and kinetic privileging of specific regions within an existing ensemble. In this sense, the conformational landscape of STING is permissive by default, and ligand binding acts not by opening a forbidden pathway [56], but by reshaping how the system navigates an already accessible space.

We note that our use of multiple independent unbiased MD simulations was chosen deliberately rather than as an alternative to enhanced sampling. Enhanced sampling approaches can efficiently accelerate conformational exploration and have been successfully applied to characterize protein free-energy landscapes and conformational transitions [57]. However, these methods generally require predefined collective variables or bias potentials. For STING LBD activation, which involves coupled α1-helix separation, dimer reorientation, and long-range communication-network remodeling, defining a small set of optimal collective variables is challenging and may introduce coordinate-dependent sampling bias. Because our analysis relies on MSM-based estimation of conformational populations, transition times, and timescale separation, preserving unbiased dynamic trajectories on the native potential energy surface was essential. Therefore, the multistart unbiased simulation design provided a more appropriate basis for comparing apo and ligand-bound ensembles under identical dynamic conditions.

Within this permissive landscape, ligand binding exerts its regulatory effect by redistributing dynamic weight through selective reorganization of how motions are coupled across the structure, rather than by globally suppressing motion [58]. C-di-GMP binding selectively dampens nonproductive fluctuations while preserving collective motions associated with α1-helix reorientation, thereby biasing the system toward activation-relevant modes without rigidifying the structure. This distinction is critical: activation is not achieved by immobilization, but by filtering dynamics such that remaining motions are more likely to drive productive conformational transitions.

At the ensemble level, this redistribution of dynamic coupling manifests as a reweighting of thermodynamic stability and transition kinetics. In the absence of ligand, STING preferentially occupies highly expanded conformations and exhibits slow, distributed interstate transitions. Ligand binding suppresses these extreme states and stabilizes intermediate conformations, while simultaneously focusing transition pathways through kinetically privileged regions of conformational space. This coupled thermodynamic–kinetic reorganization converts a dispersed and permissive ensemble into one that is biased toward activation, without eliminating conformational flexibility.

Crucially, this ensemble-level reweighting is implemented through a reorganization of the internal interaction network. Similar long-range effects have been observed in other systems, where either distal sequence variations or small-molecule allosteric modulators reshape functional interfaces and receptor signaling through propagated conformational perturbations rather than direct disruption of canonical binding contacts [59,60]. Ligand binding restructures the coupling architecture among functional regions, transforming a diffuse and weakly coordinated network into a more hierarchical topology [61] with selectively reinforced interregional interactions. This network remodeling provides the structural substrate through which energetic and kinetic biases are imposed and maintained, ensuring that conformational fluctuations are coordinated across the dimer rather than dissipated locally.

The functional consequence of this network reorganization is a focusing of allosteric information flow, which acts as the immediate mechanistic link between interaction architecture and activation-relevant conformational dynamics. In the apo state, signal propagation relies on dispersed, interface-dependent routes that are redundant and circuitous. Upon ligand binding, these routes collapse into shorter, more direct pathways that are predominantly intrachain and probabilistically dominant. By reducing path redundancy and concentrating communication through a limited set of high-fidelity routes, ligand binding enhances the efficiency and reliability of long-range signal transmission, aligning information flow with activation-relevant motions.

It should be noted that the present analyses establish coordinated relationships among ligand-induced conformational reweighting, dynamic coupling reorganization, and information-flow focusing, but do not by themselves provide direct perturbation-based proof of causality. In particular, we did not mutate residues located along the dominant communication pathways or computationally perturb specific high-coupling edges to test their effects on macrostate populations or pathway distributions. Therefore, the proposed mechanism should be interpreted as an integrated, data-supported model linking ensemble reweighting and communication-network remodeling, rather than as a definitive causal demonstration. Future residue-level perturbation, mutational simulations, or edge-perturbation analyses will be valuable for testing whether specific communication routes are necessary for the ligand-induced redistribution of STING conformational states.

Taken together, our results support a view in which allosteric activation is not achieved through discrete structural locking, but emerges from ligand-induced reprogramming of interaction architecture that focuses internal information flow, thereby reshaping conformational energetics and kinetics across a physically accessible ensemble. In STING, ligand binding does not create new structural states or eliminate conformational flexibility; rather, it redistributes probabilities, reorganizes transition pathways, and constrains information flow such that remaining motions are selectively aligned with activation-relevant directions. This perspective reconciles static structural snapshots with dynamic signaling behavior and emphasizes that effective allosteric regulation operates by biasing ensemble organization rather than enforcing unique conformations. More broadly, these findings suggest that targeting the energetic and informational organization of protein ensembles—rather than individual static structures—may represent a general and powerful strategy for modulating allosteric signaling in complex biological systems.

## Data Availability

The trajectory data supporting the findings of this study have not been deposited in a public repository but are available from the corresponding authors upon reasonable request. Other data needed to evaluate the conclusions of this study are available in the paper and/or the Supplementary Materials.
