# Supplementary material for: Allosteric Activation through Coordinated Energy Landscape Reweighting and Information Flow
Source: Comput Struct Biotechnol J. 2026 Jun 9;35(1):0133. doi: 10.34133/csbj.0133 (PMC13247314; doi:10.34133/csbj.0133)
Supplement: Supplementary 1 — Figs. S1 to S13 [file csbj.0133.f1.zip › Supplementary Information .docx]

**
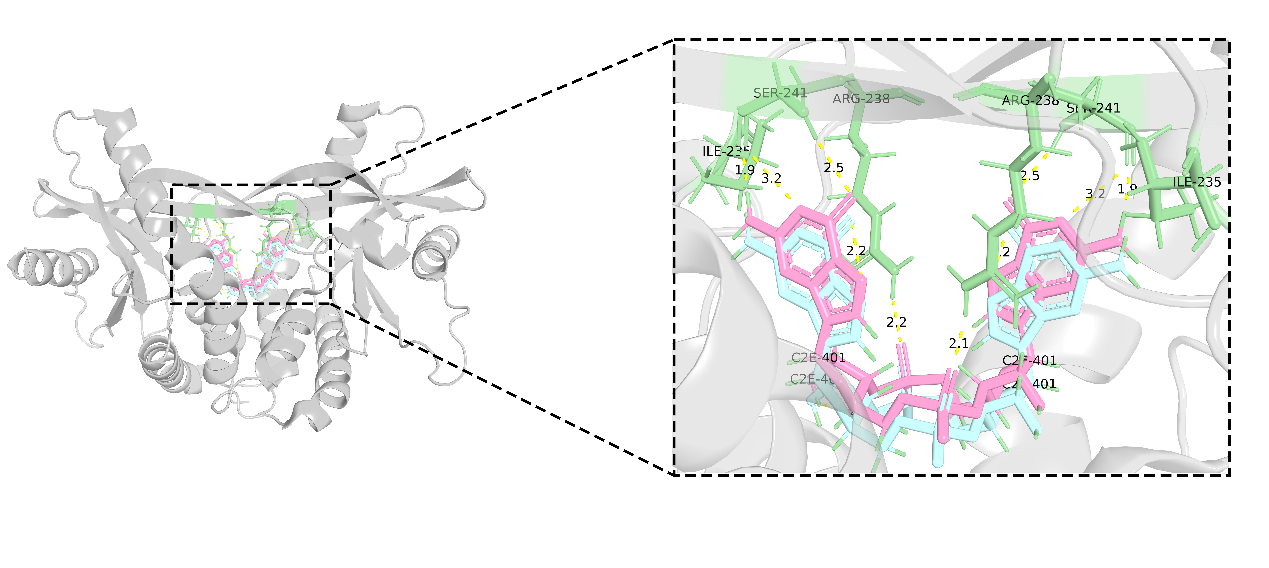
**

**Figure S1. Validation of the docked C-di-GMP pose against the crystallographic binding mode in STING LBD.**Superposition of the docked C-di-GMP pose and the experimentally resolved crystallographic C-di-GMP pose from the 4F5D ligand-bound STING LBD structure. The STING LBD is shown in gray cartoon representation, and the ligand-binding pocket is highlighted in the enlarged view. The crystallographic and docked C-di-GMP poses are shown in pink and cyan, respectively, after least-squares alignment of the protein backbones. The ligand heavy-atom RMSD between the docked and crystallographic C-di-GMP poses is 0.830 Å, indicating close agreement between the docked pose and the experimental binding mode. Key binding-pocket residues, including Ile235, Arg238, and Ser241, are shown in green stick representation. Yellow dashed lines indicate representative polar contacts or short-range ligand–protein interactions, with distances labeled in Å. The docked C-di-GMP remains located in the canonical STING LBD ligand-binding pocket and preserves the overall ligand orientation and major binding-pocket interaction features observed in the crystallographic structure. This comparison supports the reliability of the docked C-di-GMP pose used to construct ligand-bound intermediate and open-like conformations lacking experimentally resolved ligand-bound structures.

**
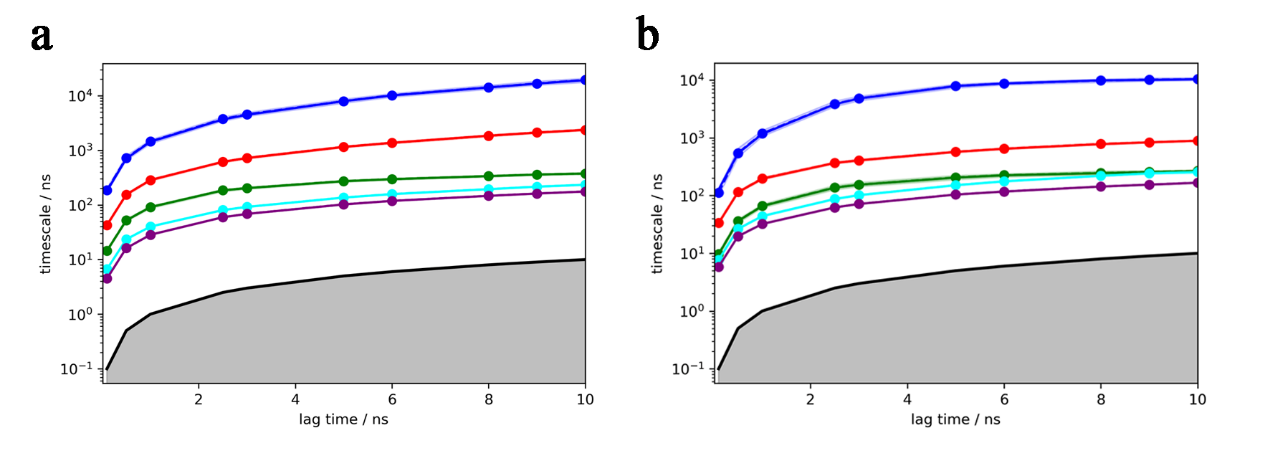
**

**Figure S2. Implied timescales analysis for the two systems.**

**(a)** C-di-GMP-bound system.
**(b)** Unbound system.

Implied timescales as a function of lag time were calculated using Bayesian Markov state models constructed from 1000 microstates. Solid lines represent the dominant implied timescales, and shaded regions indicate the corresponding 95% credible intervals estimated from posterior sampling. The black line denotes the condition $t=\tau$, and the gray shaded region corresponds to timescales shorter than the lag time, where the Markov assumption is not valid.

For both systems, the slowest implied timescales are well separated from the lag time across the examined range, indicating that the Markovian assumption is satisfied. In addition, the first few dominant timescales exhibit clear convergence (plateau behavior) at lag times above ~5 ns, supporting the choice of lag time used for MSM construction. The presence of a clear spectral gap between the leading timescales further justifies the coarse-graining into three metastable macrostates. The narrow uncertainty bands indicate that the estimated timescales are statistically robust.


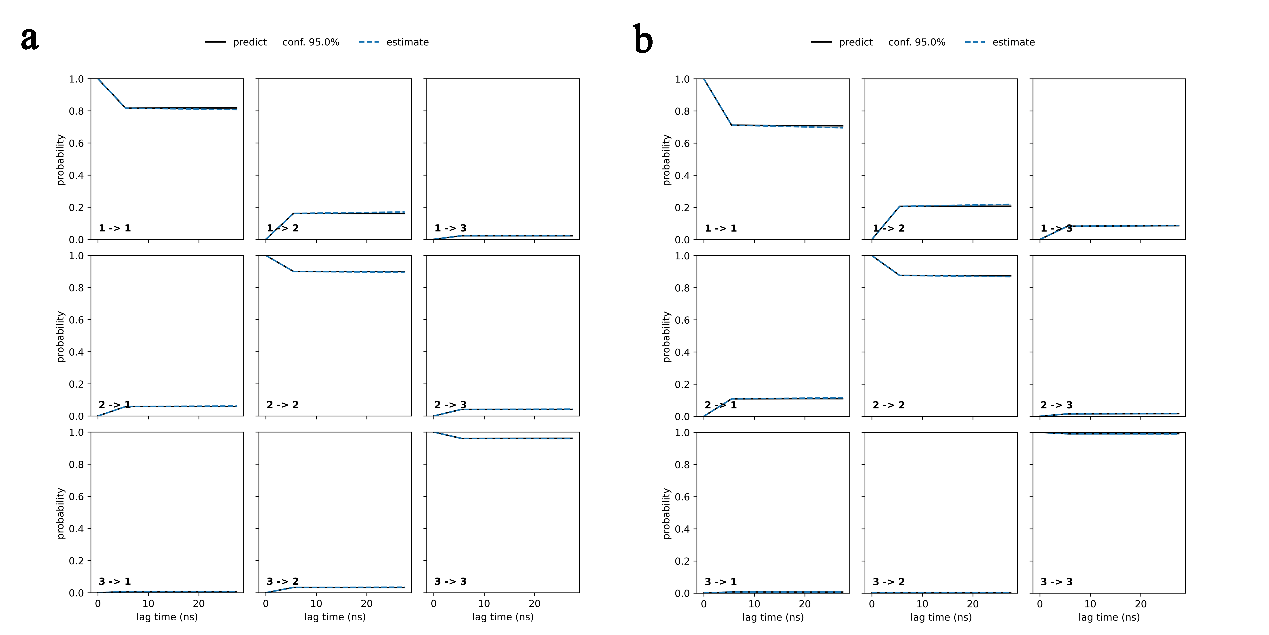


**Figure S3. Chapman–Kolmogorov validation of the three-state MSMs for the two systems.**

**(a)** C-di-GMP-bound system.
**(b)** Unbound system.

Chapman–Kolmogorov (CK) tests for the three-state Markov state models constructed for the C-di-GMP-bound and unbound systems. Solid lines represent the transition probabilities predicted by the MSMs, whereas dashed lines denote the corresponding direct estimates from the simulation data.

For both systems, the predicted and observed transition probabilities agree closely across all state pairs and lag-time multiples, demonstrating that the MSMs accurately reproduce the time evolution of the systems at the chosen lag time. This agreement supports the validity of the Markovian approximation and confirms the reliability of the three-state coarse-grained models used for subsequent kinetic and thermodynamic analyses.


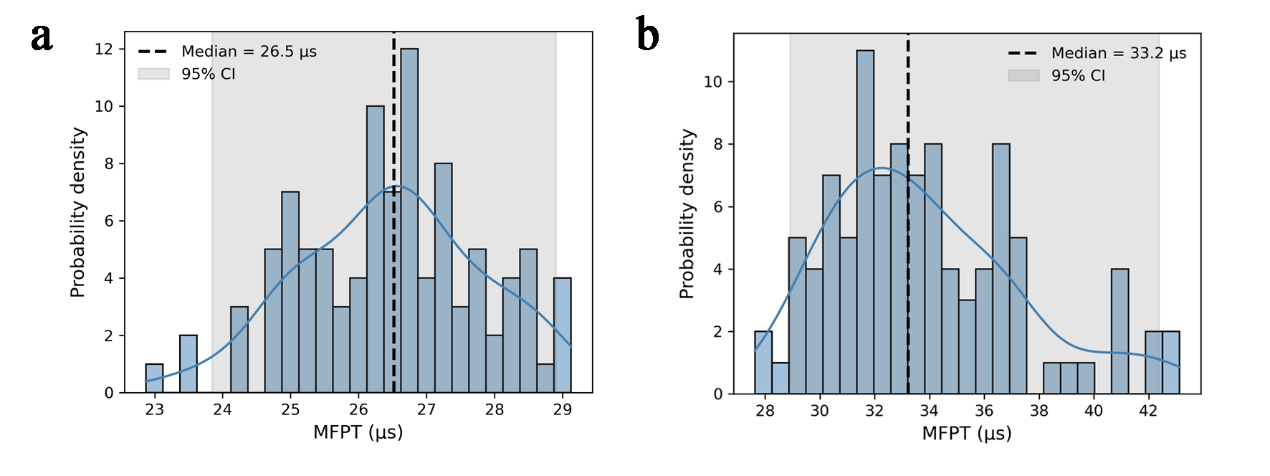


**Figure S4. Posterior distributions of the slowest mean first passage times for the two systems.**

**(a)** C-di-GMP-bound system.
**(b)** Unbound system.

Posterior distributions of the mean first passage time (MFPT) for the slowest macrostate transition, estimated from Bayesian Markov state models for the C-di-GMP-bound and unbound systems. The histograms summarize the distribution of MFPT values obtained from posterior sampling, thereby quantifying the statistical uncertainty of the kinetic estimates.

For both systems, the MFPT distributions are unimodal, indicating that the inferred slow timescales are statistically well defined rather than arising from unstable or poorly sampled models. The C-di-GMP-bound system shows MFPTs on the order of ~20–30 μs, whereas the unbound system exhibits slightly slower kinetics, with MFPTs centered at ~30–40 μs. The relatively narrow distributions support the robustness of the reported microsecond-scale transition dynamics.


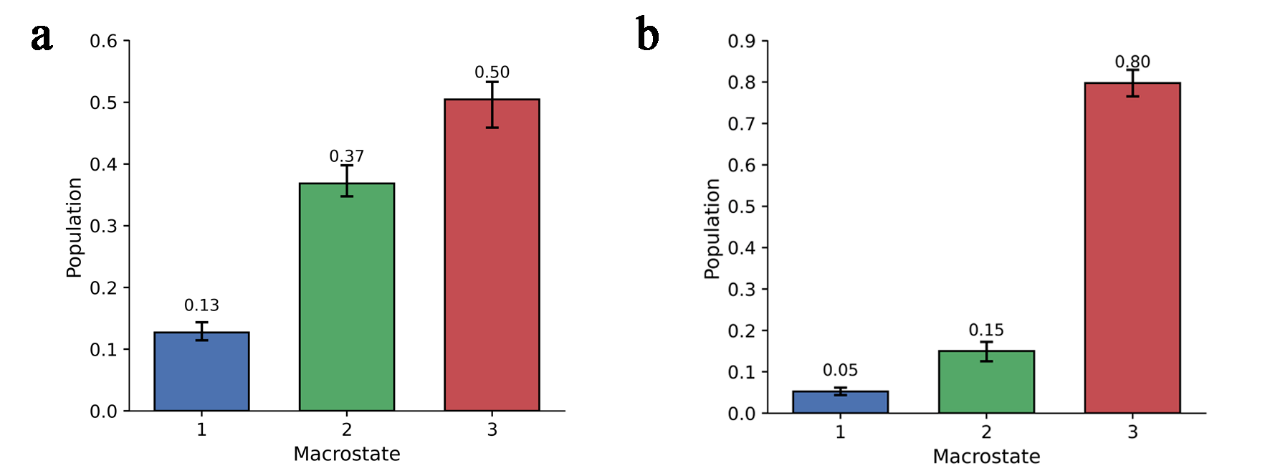


**Figure S5. Equilibrium populations of the three macrostates for the two systems.**

**(a)** C-di-GMP-bound system.
**(b)** Unbound system.

Equilibrium populations of the three macrostates estimated from the Bayesian Markov state models for the C-di-GMP-bound and unbound systems. Bars represent the mean macrostate populations, and error bars denote the 95% credible intervals obtained from posterior sampling.

The C-di-GMP-bound system exhibits a more heterogeneous conformational ensemble, with populations distributed across all three macrostates. In contrast, the unbound system is dominated by a single macrostate, while the other two states are only weakly populated. The narrow error bars indicate that the macrostate populations are statistically stable in both systems. These results support a more balanced free-energy landscape in the C-di-GMP-bound system and a more strongly biased energy landscape in the unbound system.

**
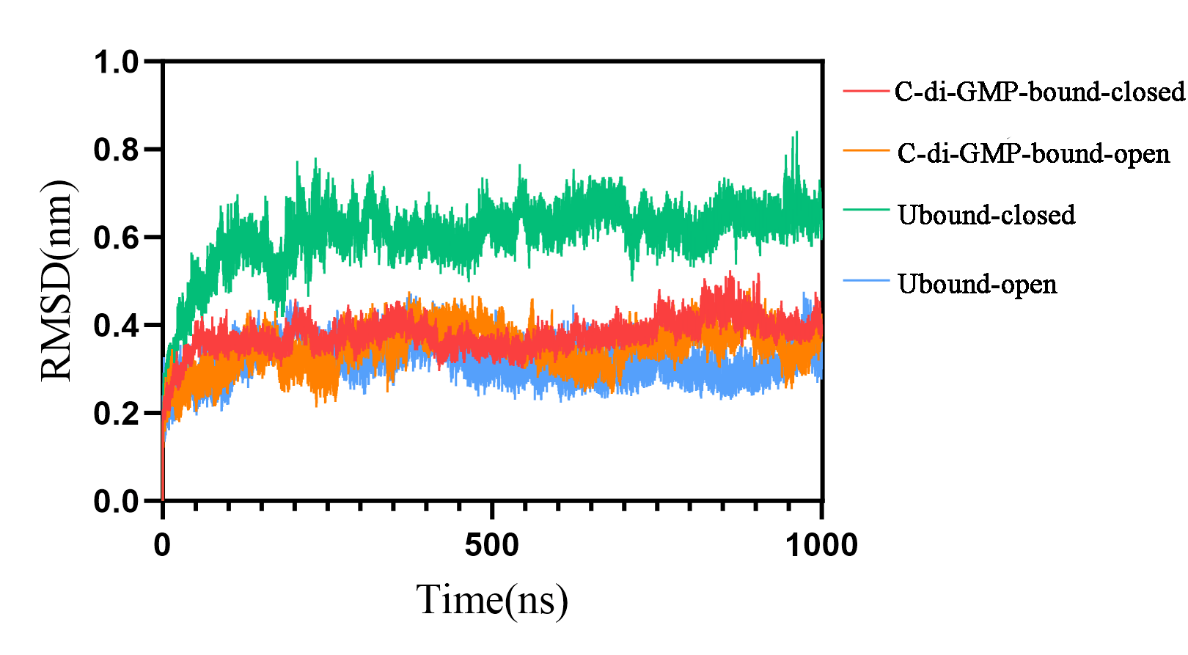
**

**Figure S6. Endpoint-referenced RMSD analysis of simulated STING LBD conformations.**

Backbone RMSD profiles of the apo and C-di-GMP–bound STING LBD trajectories calculated relative to the experimentally resolved closed and open structures. The uniformly processed closed structure (PDB: 4F5D) and open structure (PDB: 4F5Y), containing residues 153–340 of chains A and B, were used as endpoint references. Before RMSD calculation, trajectory frames were aligned to the STING LBD backbone.These endpoint-referenced RMSD profiles complement the initial-structure-referenced RMSD analysis shown in Fig. 3 and provide a direct comparison between simulated conformations and the canonical closed and open endpoint structures.

**
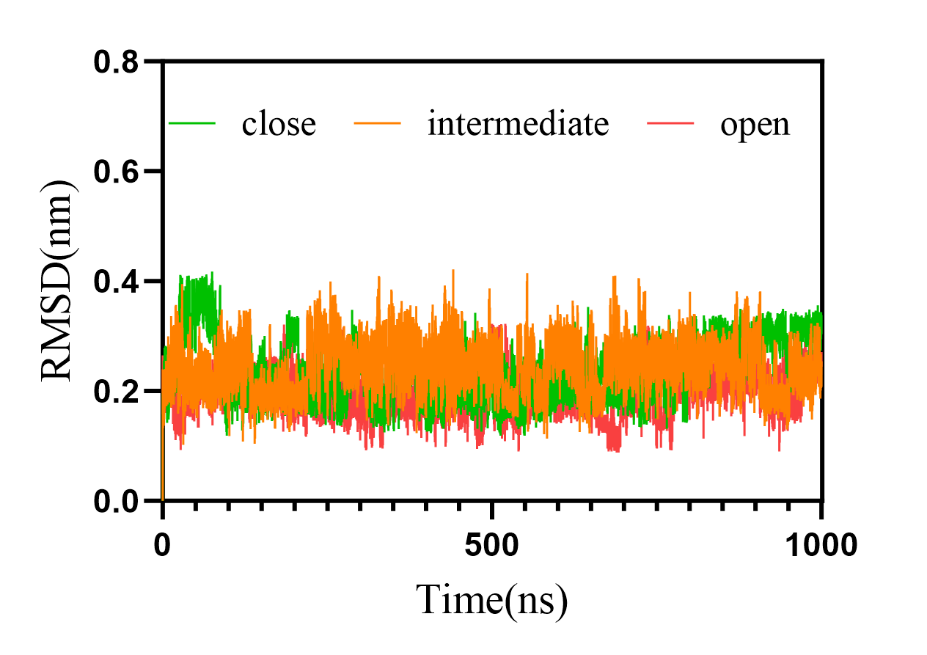
**

**Figure S7. Stability of C-di-GMP in representative STING–C-di-GMP complex simulations.**Heavy-atom RMSD profiles of C-di-GMP calculated from three representative ligand-bound trajectories initiated from the closed conformation, one intermediate conformation, and the open conformation of the STING LBD. For each trajectory, RMSD was calculated after least-squares alignment to the protein backbone, using the initial ligand-bound structure of the corresponding trajectory as the reference. The RMSD profiles therefore reflect the positional stability of C-di-GMP relative to the STING LBD binding pocket. C-di-GMP remained stably bound in all three representative simulations, and no positional restraints were applied to the ligand during production MD.

**
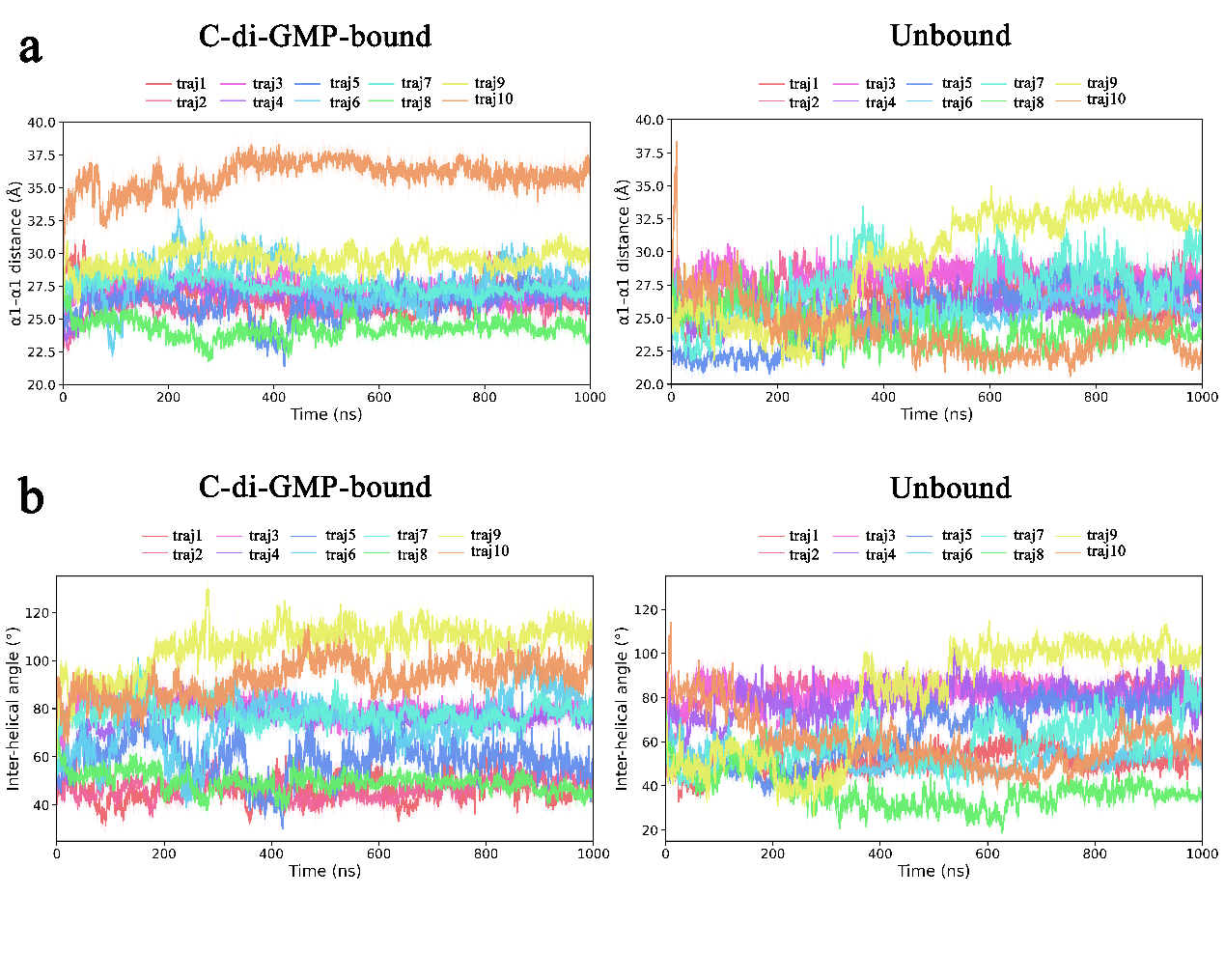
**

**Figure S8. Time evolution of α1–α1 distance and inter-helical angle across all independent trajectories.**The α1–α1 distance and inter-helical angle were monitored as functions of simulation time for all ten independent trajectories in both the C-di-GMP-bound and unbound systems. The α1–α1 distance was calculated using the geometric centers of the two α1 helices. The inter-helical angle was calculated from the corresponding helix-axis vectors. In each panel, different colors represent independent trajectories. The time-series profiles show that both structural descriptors sampled broad conformational ranges across the simulation ensemble rather than being restricted to a single representative trajectory. These results provide trajectory-level evidence that compact, intermediate, and expanded α1 arrangements were sampled in both systems.

**
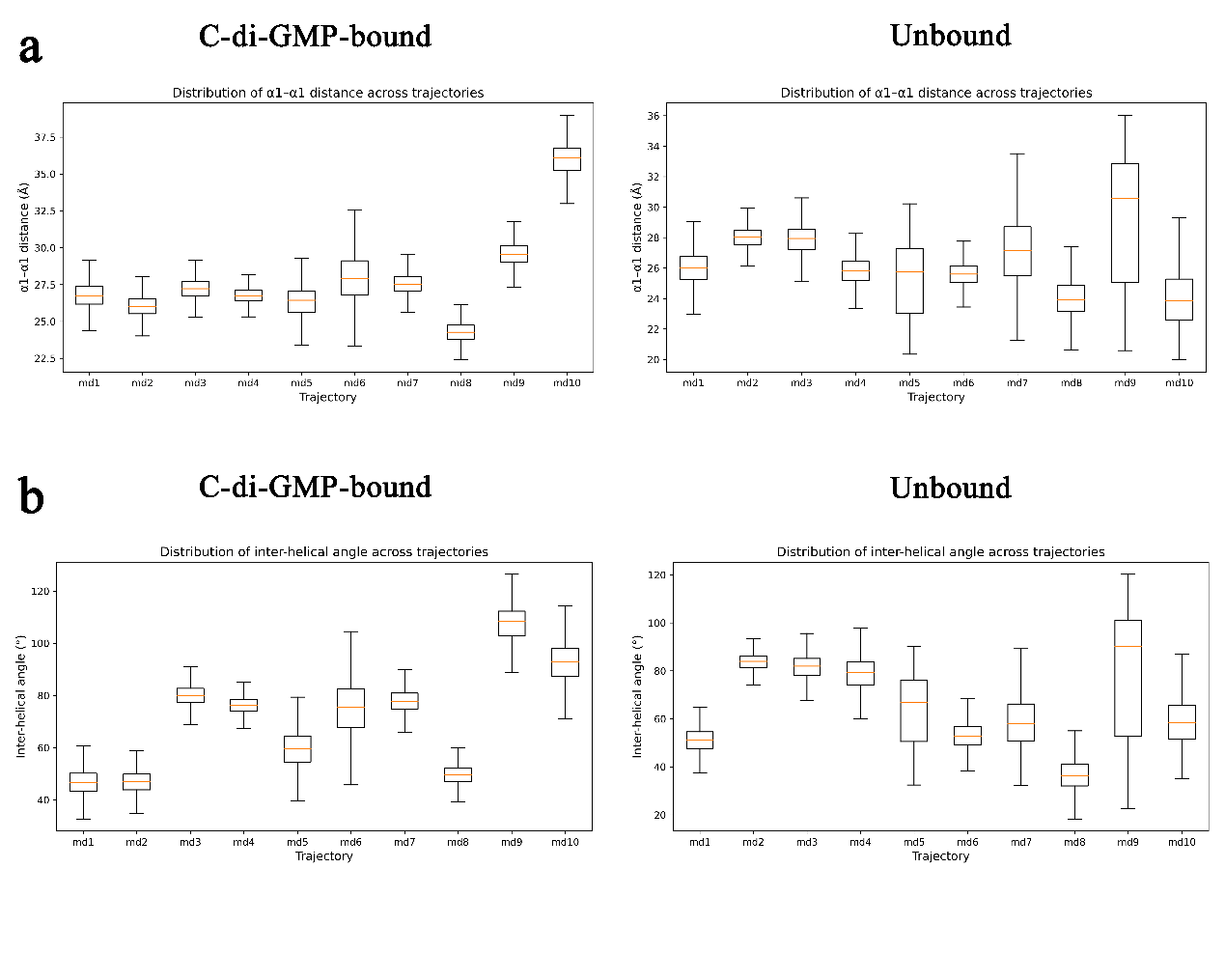
**

**Figure S9. Trajectory-wise distributions of α1–α1 distance and inter-helical angle.**Boxplots summarize the distributions of α1–α1 distance and inter-helical angle for each of the ten independent trajectories in the C-di-GMP-bound and unbound systems. The box represents the interquartile range, the horizontal line indicates the median, and the whiskers indicate the overall distribution range excluding extreme outliers. The distributions reveal that different trajectories sampled distinct regions of the α1 conformational space. In both systems, the α1–α1 distance and inter-helical angle covered compact, intermediate, and expanded conformational regimes, indicating that the conformational heterogeneity observed in the simulations was not driven by a single trajectory. Together with the time-series analyses in Figure S8, these results support ensemble-level sampling of the key α1-mediated structural descriptors.


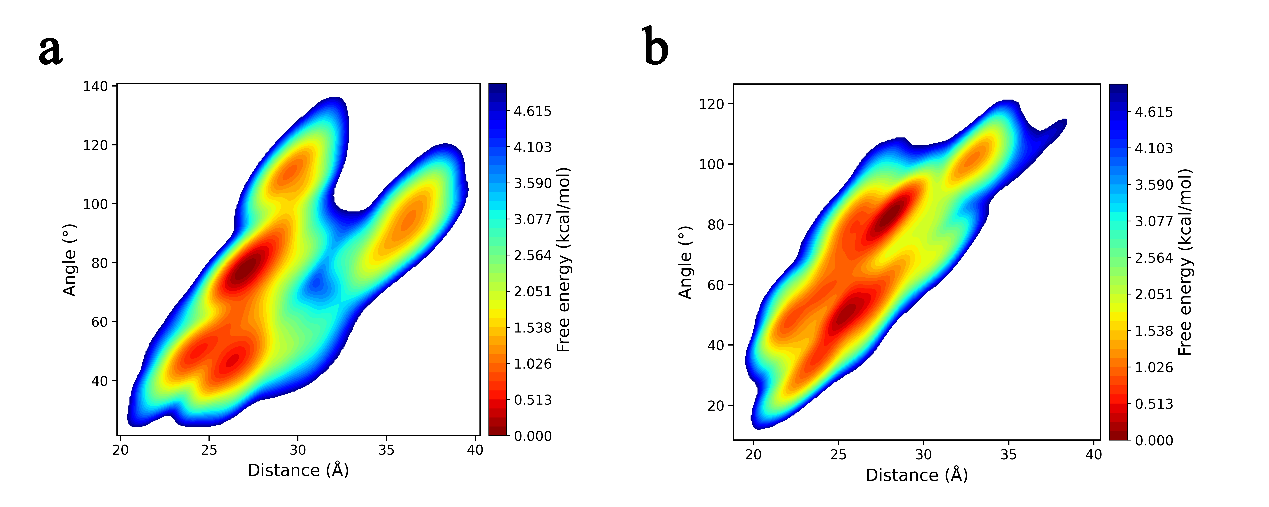


**Figure S10. Free-energy landscapes reconstructed from pooled trajectories using α1–α1 distance and inter-helical angle as reaction coordinates.**
Two-dimensional free-energy landscapes were reconstructed by pooling all ten independent trajectories for each system and projecting the conformational ensembles onto the α1–α1 distance and inter-helical angle coordinates. The relative free energy was calculated from the probability density distribution and is shown in kcal/mol. Warmer colors indicate lower free-energy regions, whereas cooler colors indicate higher free-energy regions. Multiple low-energy basins are observed in both the C-di-GMP-bound(a) and unbound systems(b), corresponding to structurally distinct α1 arrangements. These pooled free-energy landscapes demonstrate that the conformational regions discussed in the manuscript are supported by the full trajectory ensemble rather than by a single representative trajectory.


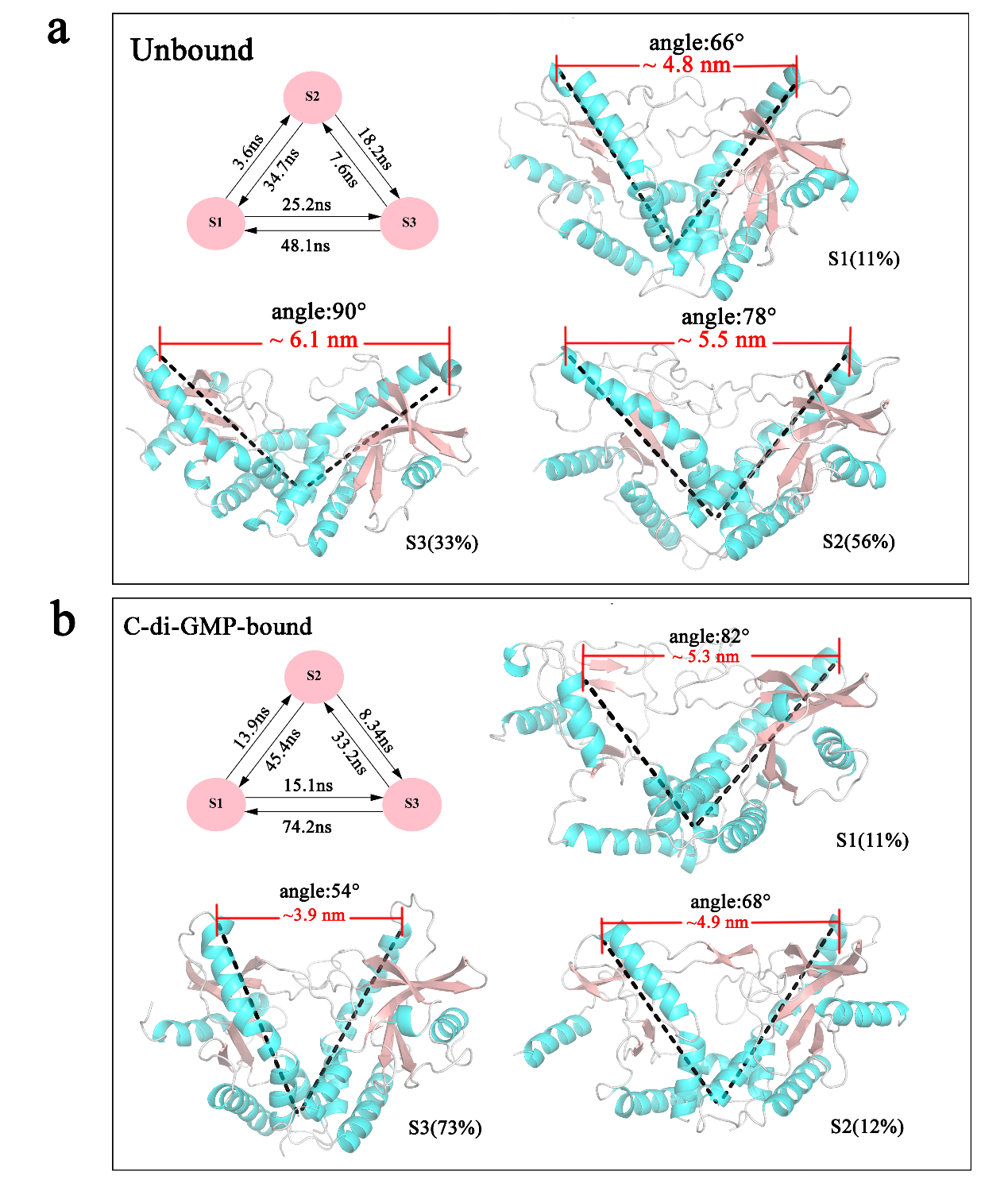


**Figure S11. Feature-robustness analysis using interface-contact-based MSMs.**

Alternative MSMs were constructed using inter-chain interface contact features to assess whether the ligand-dependent conformational reweighting observed in the primary β2-distance-based MSM depends on the choice of input features.
**(a)** Macrostate transition network and representative structures of the unbound STING LBD obtained from the interface-contact-based MSM. Three macrostates were identified. The unbound ensemble is distributed across expanded conformations, with S2 representing the dominant state (56%; α1–α1 distance ~5.5 nm, inter-helical angle ~78°), followed by S3 (33%; ~6.1 nm, ~90°) and S1 (11%; ~4.8 nm, ~66°).
**(b)** Corresponding macrostate transition network and representative structures of the C-di-GMP-bound STING LBD. In contrast to the unbound system, the ligand-bound ensemble is strongly enriched in the more compact S3 state (73%; α1–α1 distance ~3.9 nm, inter-helical angle ~54°), whereas the more expanded S1 and S2 states account for 11% and 12% of the population, respectively. Arrows in the transition networks indicate dominant inter-macrostate transitions, with labels reporting MSM-inferred mean first passage times. Representative structures are shown with α-helices in cyan, β-strands in pink, and loop regions in gray; red annotations indicate the α1–α1 separation distance and inter-helical angle. The interface-contact-based MSM reproduces the qualitative ligand-dependent conformational reweighting observed in the primary β2-distance-based model, supporting that the conclusion of C-di-GMP-induced suppression of highly expanded conformations and stabilization of compact/intermediate regimes is robust to feature choice.


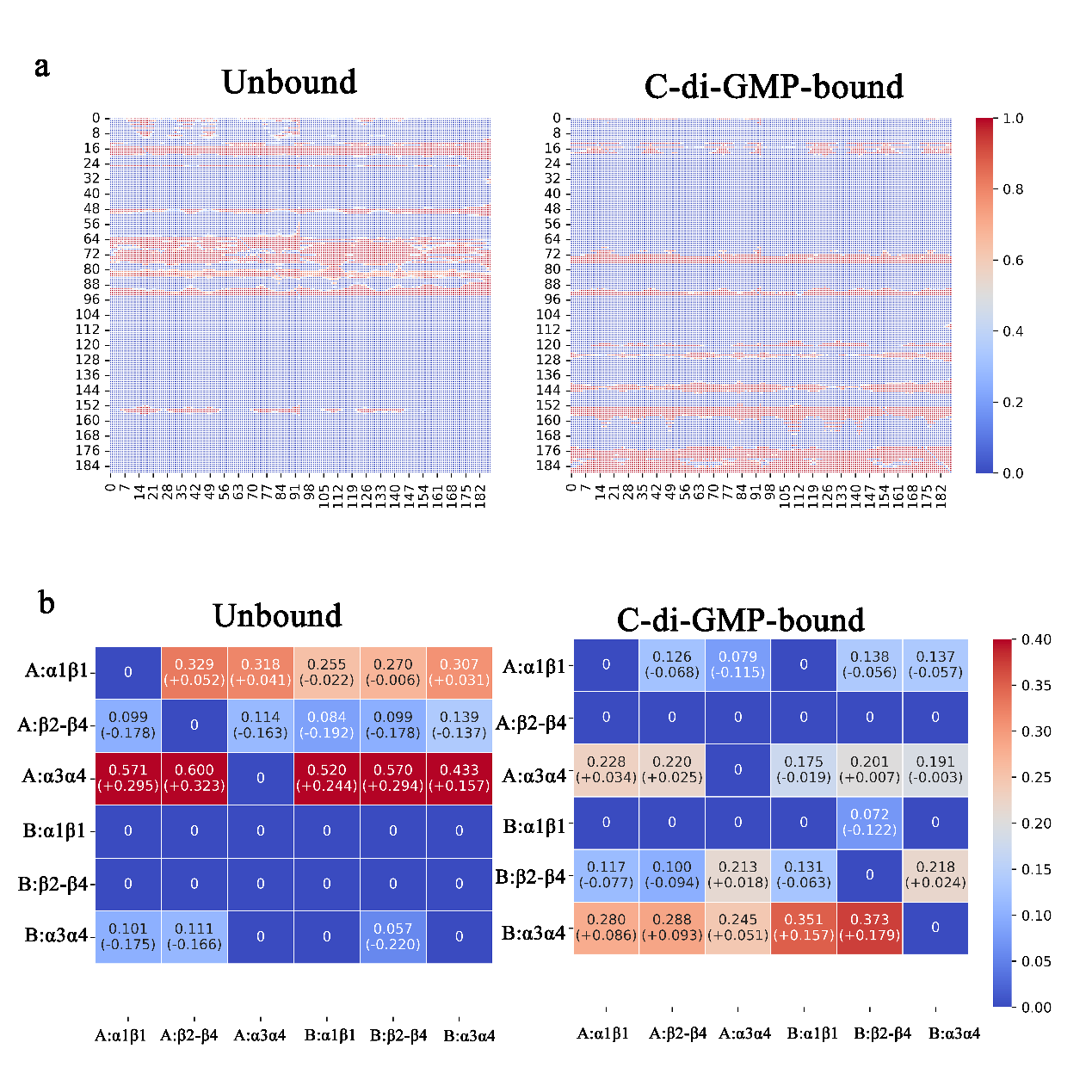


**Figure S12. Threshold-sensitivity analysis of NRI-inferred dynamic coupling networks using an edge-probability cutoff of 0.1.**

NRI-inferred dynamic coupling networks were reconstructed using a relaxed edge-probability threshold of 0.1 to evaluate whether the network topology depends on the 0.2 cutoff used in the primary analysis.
**(a)** Residue-level NRI coupling maps for the unbound and C-di-GMP–bound STING LBD systems under the 0.1 threshold. Color intensity represents inferred edge probability or coupling strength, with higher values indicating stronger dynamical coupling.
**(b)** Region-averaged coupling matrices based on the same six functional regions used in Fig. 6: A:α1β1, A:β2–β4, A:α3α4, B:α1β1, B:β2–β4, and B:α3α4. Matrix values indicate averaged interregional coupling strengths under the 0.1 threshold. Values in parentheses indicate the change relative to the primary 0.2-threshold analysis. Under the relaxed threshold, the unbound system retains a relatively diffuse coupling architecture, whereas the C-di-GMP–bound system maintains a reorganized pattern of interregional couplings. The preservation of the major apo-versus-bound differences under a more permissive cutoff supports that the ligand-induced network reorganization is not an artifact of the specific edge-probability threshold used in the primary analysis.

**
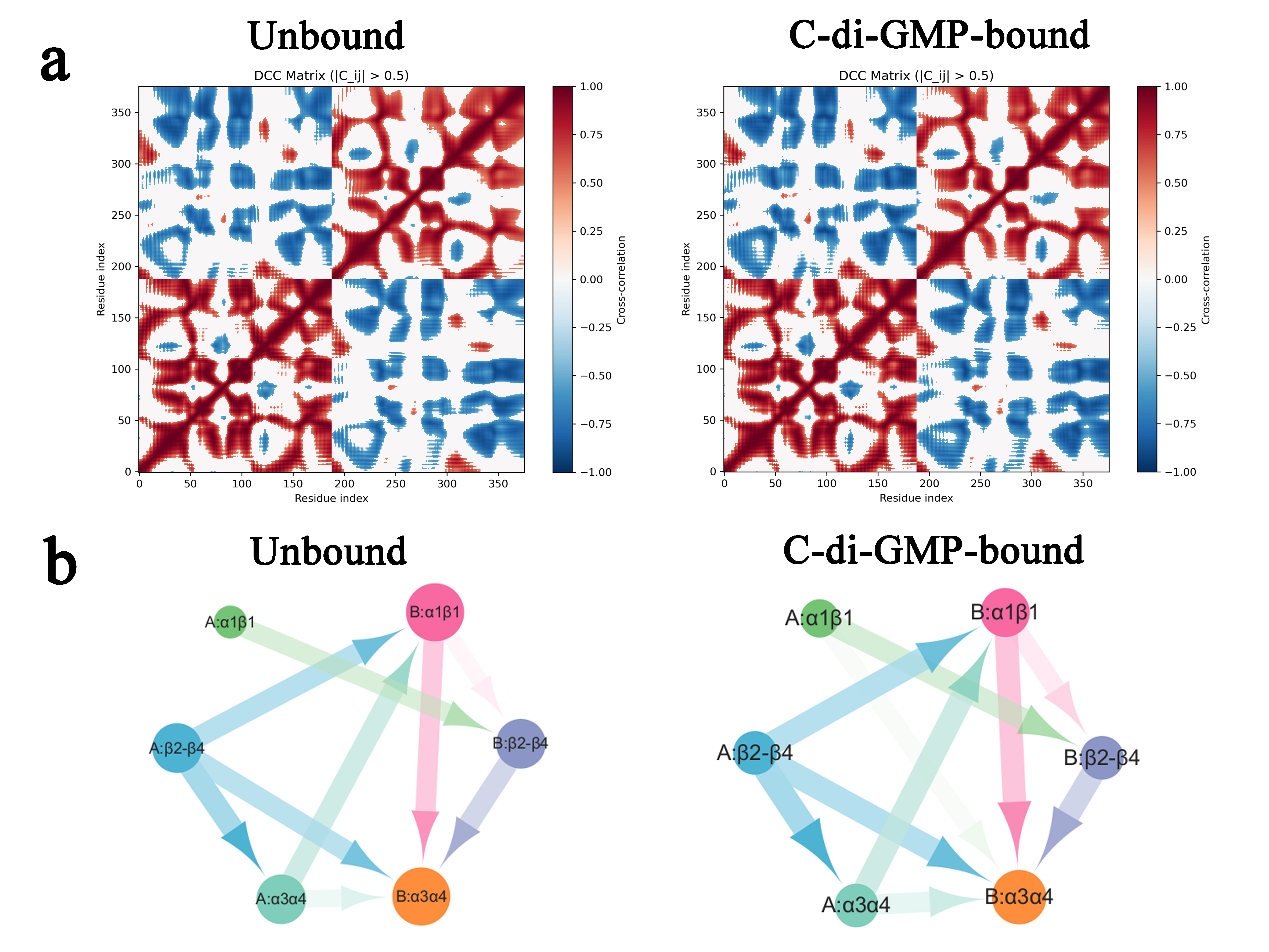
**

**Figure S13. Comparison of conventional DCCM analysis with NRI-inferred dynamic coupling networks.**

Dynamic cross-correlation matrices (DCCMs) were calculated from Cα atomic fluctuations after least-squares alignment of the MD trajectories to the STING LBD backbone. This analysis was performed as a conventional correlation-based reference to compare with the NRI-inferred interaction architecture.

**(a)** Residue-level DCCM heatmaps for the unbound and C-di-GMP–bound STING LBD systems. Correlation coefficients range from −1 to 1, with positive values indicating correlated motions and negative values indicating anti-correlated motions. For visualization, only correlations with $\mid C_{ij}\mid>0.5$are shown. Both systems display extensive correlated and anti-correlated motions across the STING LBD dimer, indicating that DCCM captures broad fluctuation correlations throughout the protein. However, the dense distribution of strong correlations makes it difficult to directly resolve specific communication routes or to identify a focused set of region-to-region coupling pathways.

**(b)** Region-level DCCM networks constructed using the same six functional regions used in the NRI analysis: A:α1β1, A:β2–β4, A:α3α4, B:α1β1, B:β2–β4, and B:α3α4. Nodes represent functional regions, and edges represent strong interregional correlations derived from the DCCM analysis. In both the unbound and C-di-GMP–bound systems, the DCCM-based regional networks remain relatively dense, with multiple strong interregional correlations retained. This indicates that DCCM is effective for detecting global correlated motions but provides limited resolution for distinguishing selective information-transfer routes or pathway focusing between the two systems.

Together, these results highlight the complementarity between DCCM and NRI. DCCM provides a conventional measure of pairwise linear fluctuation correlations, whereas NRI infers predictive dynamical dependencies from trajectory windows and yields a more structured interaction architecture suitable for identifying dominant communication routes. Thus, the DCCM comparison supports the need for NRI-based analysis to resolve ligand-induced reorganization of information flow beyond dense global correlation patterns.
