# Supplementary figures and images for: Allosteric Activation through Coordinated Energy Landscape Reweighting and Information Flow
Source: Comput Struct Biotechnol J. 2026 Jun 9;35(1):0133. doi: 10.34133/csbj.0133 (PMC13247314; doi:10.34133/csbj.0133)

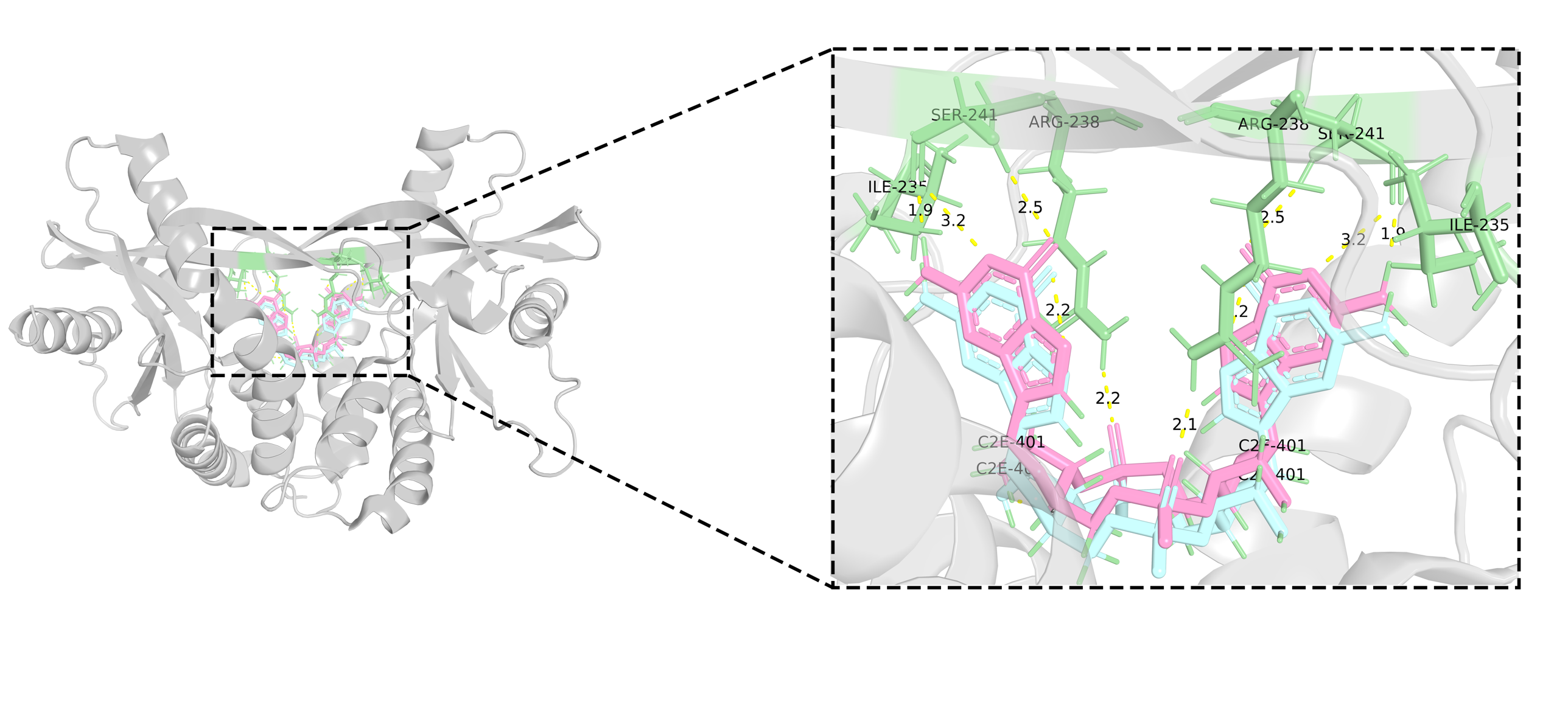

Supplement: Supplementary 1 — Figs. S1 to S13 [file csbj.0133.f1.zip › Supplementary Information-Fig/Figure S1.tif]

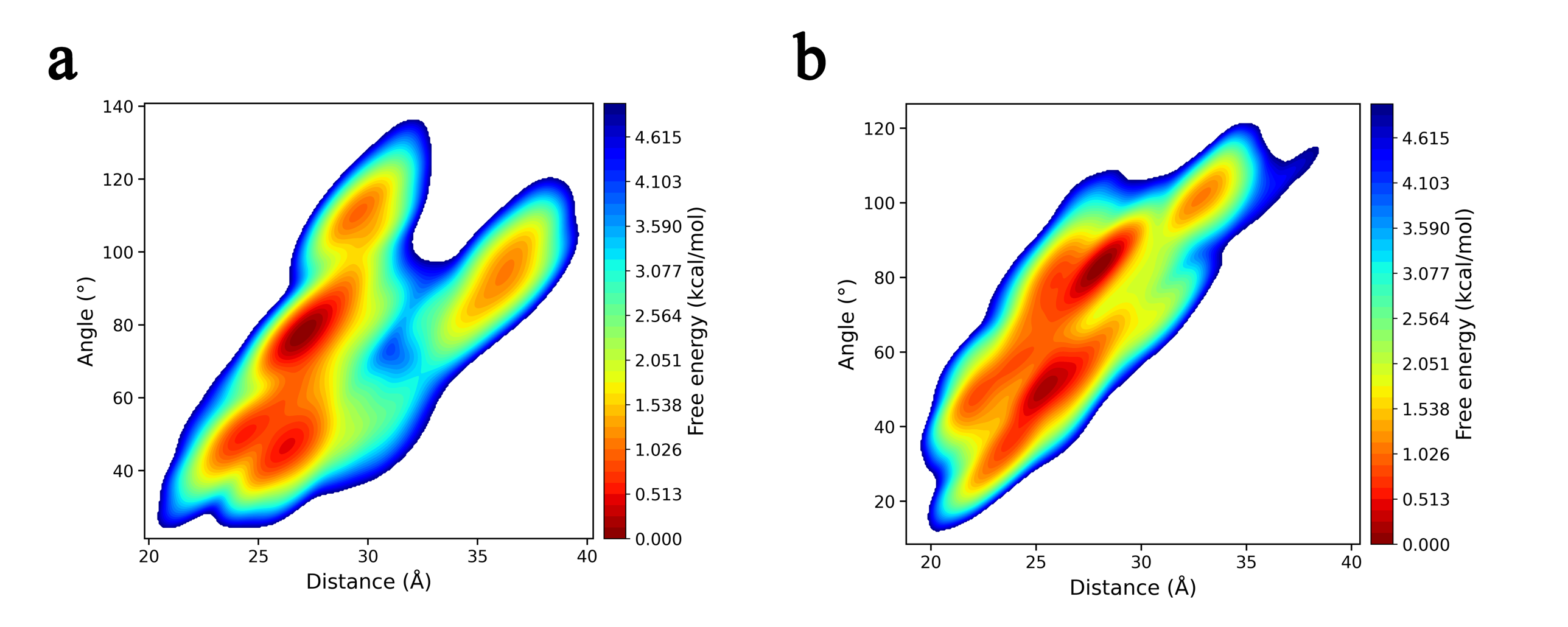

Supplement: Supplementary 1 — Figs. S1 to S13 [file csbj.0133.f1.zip › Supplementary Information-Fig/Figure S10.tif]

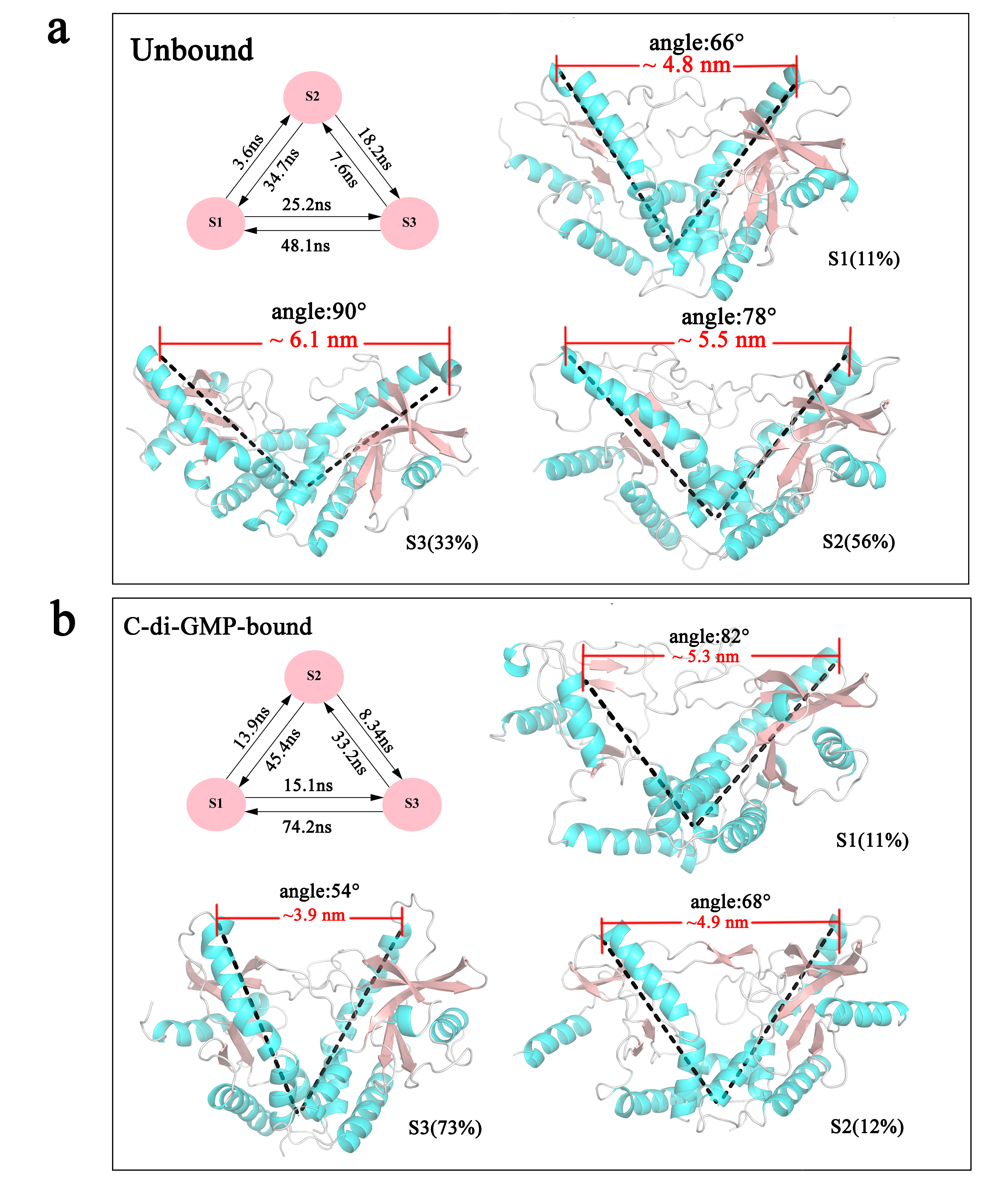

Supplement: Supplementary 1 — Figs. S1 to S13 [file csbj.0133.f1.zip › Supplementary Information-Fig/Figure S11.tif]

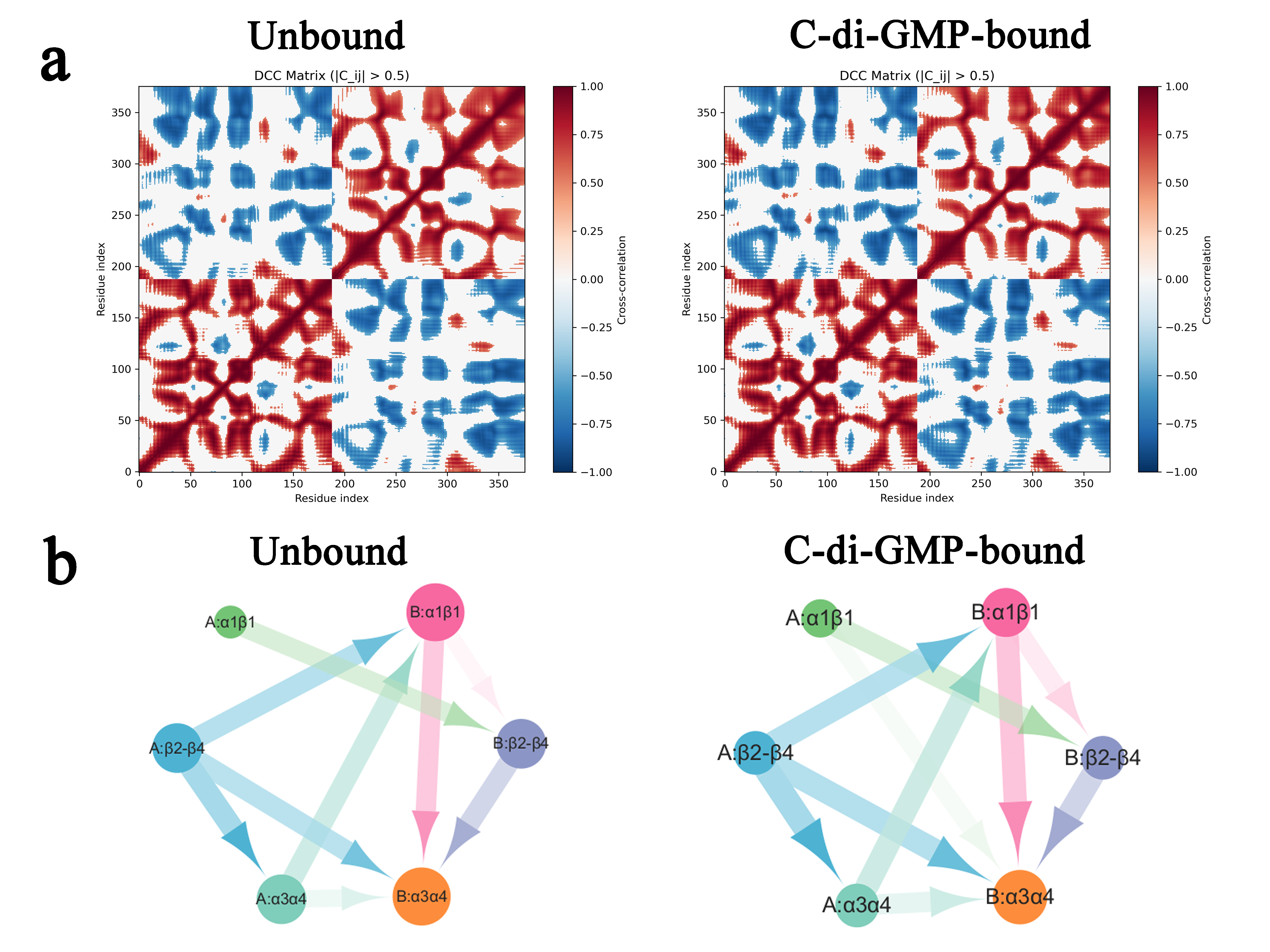

Supplement: Supplementary 1 — Figs. S1 to S13 [file csbj.0133.f1.zip › Supplementary Information-Fig/Figure S13.tif]

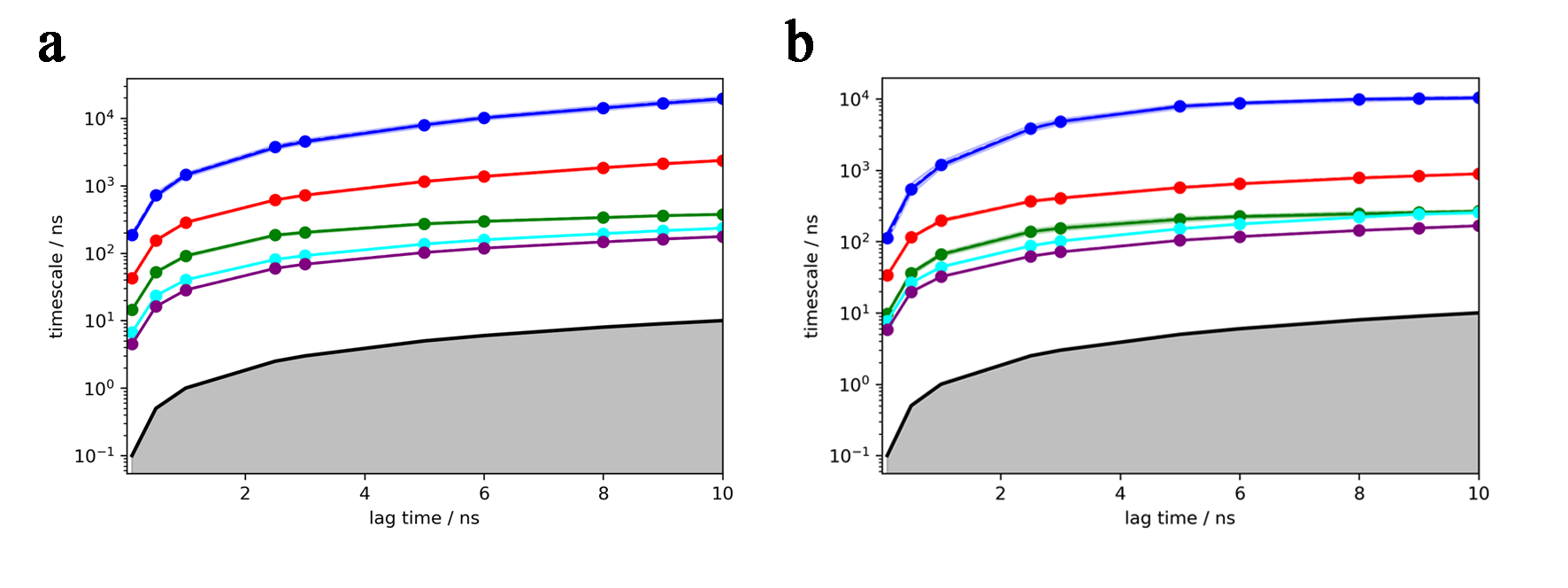

Supplement: Supplementary 1 — Figs. S1 to S13 [file csbj.0133.f1.zip › Supplementary Information-Fig/Figure S2.tif]

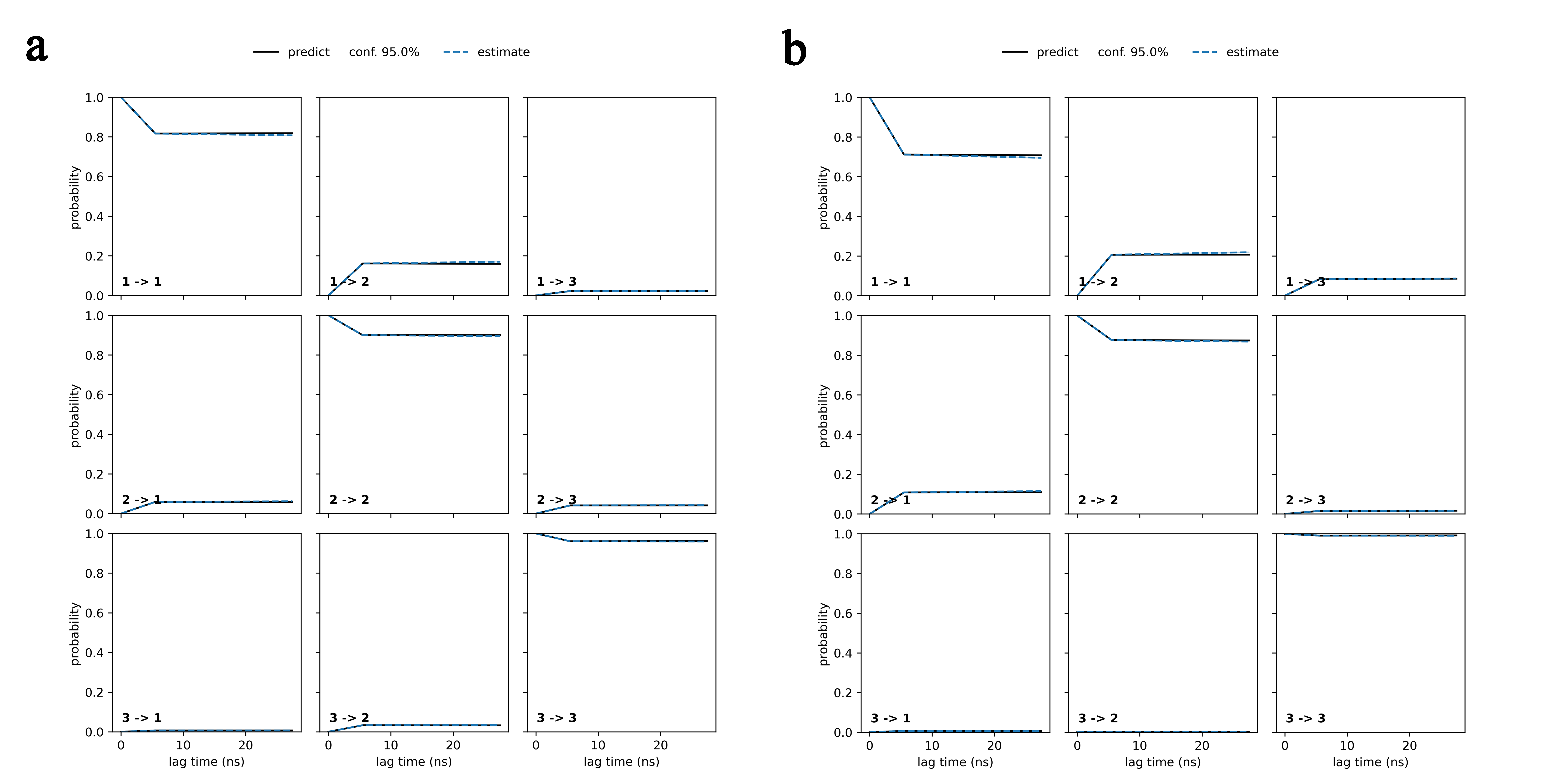

Supplement: Supplementary 1 — Figs. S1 to S13 [file csbj.0133.f1.zip › Supplementary Information-Fig/Figure S3.tif]

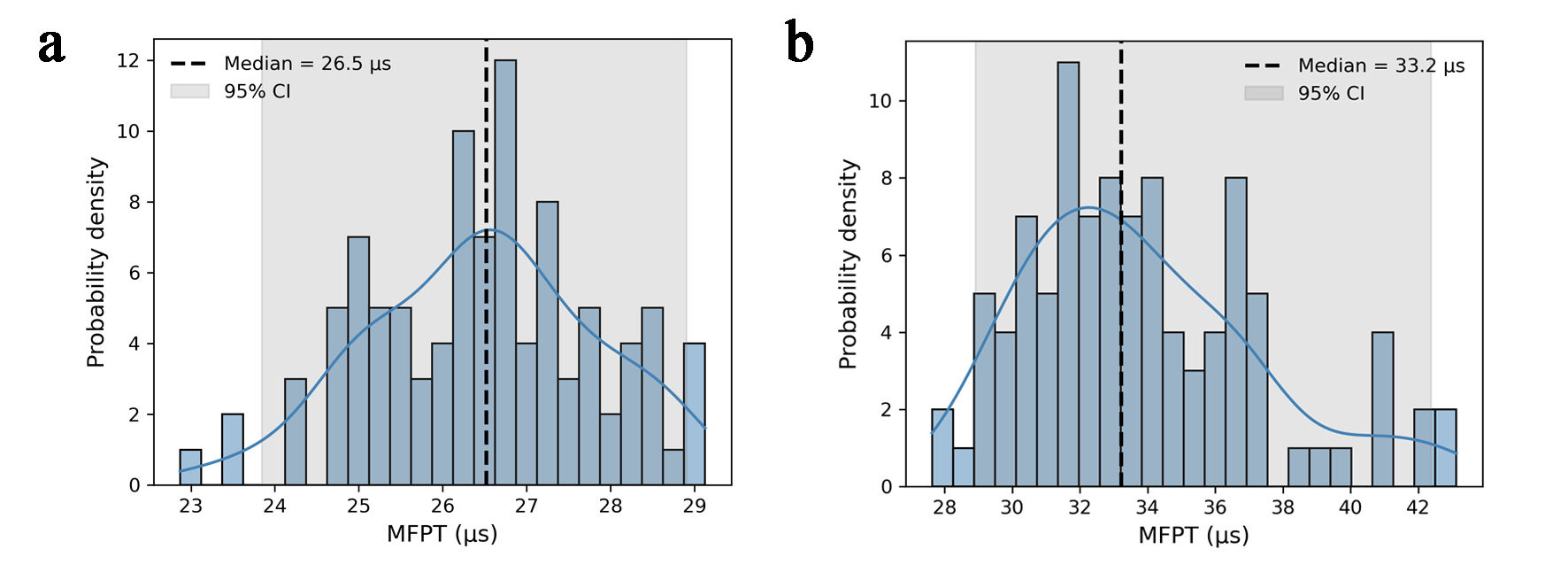

Supplement: Supplementary 1 — Figs. S1 to S13 [file csbj.0133.f1.zip › Supplementary Information-Fig/Figure S4.tif]

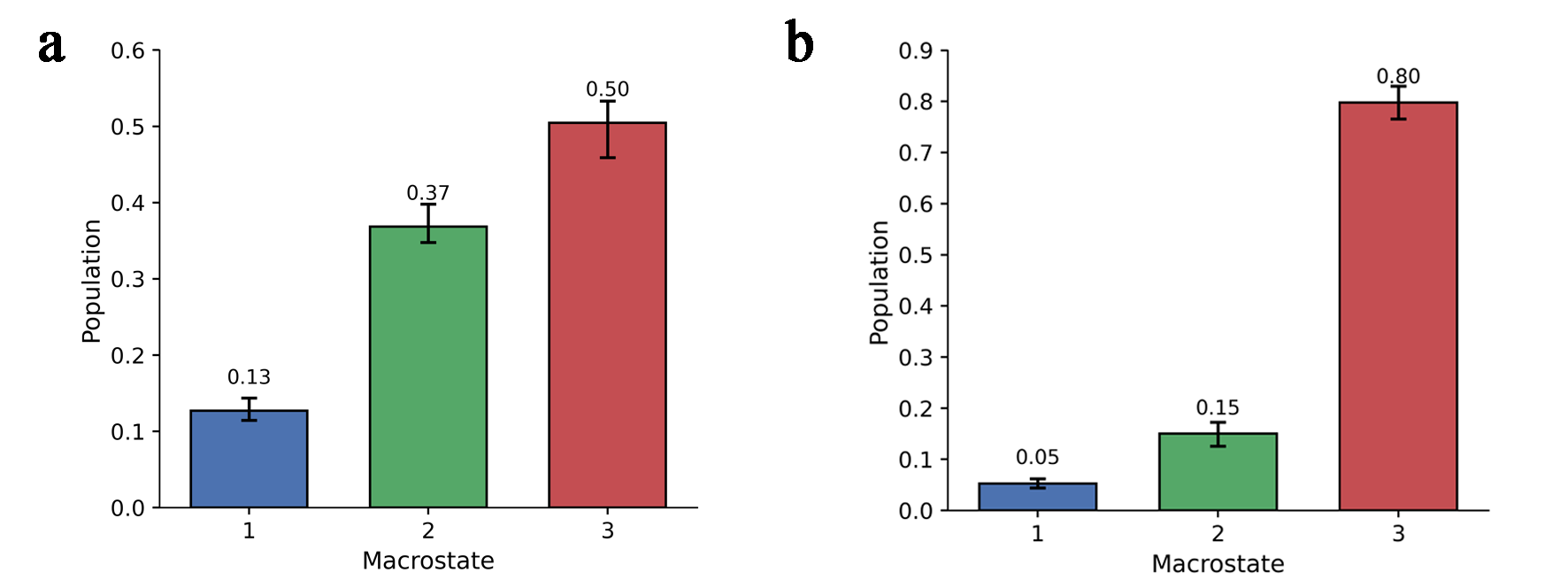

Supplement: Supplementary 1 — Figs. S1 to S13 [file csbj.0133.f1.zip › Supplementary Information-Fig/Figure S5.tif]

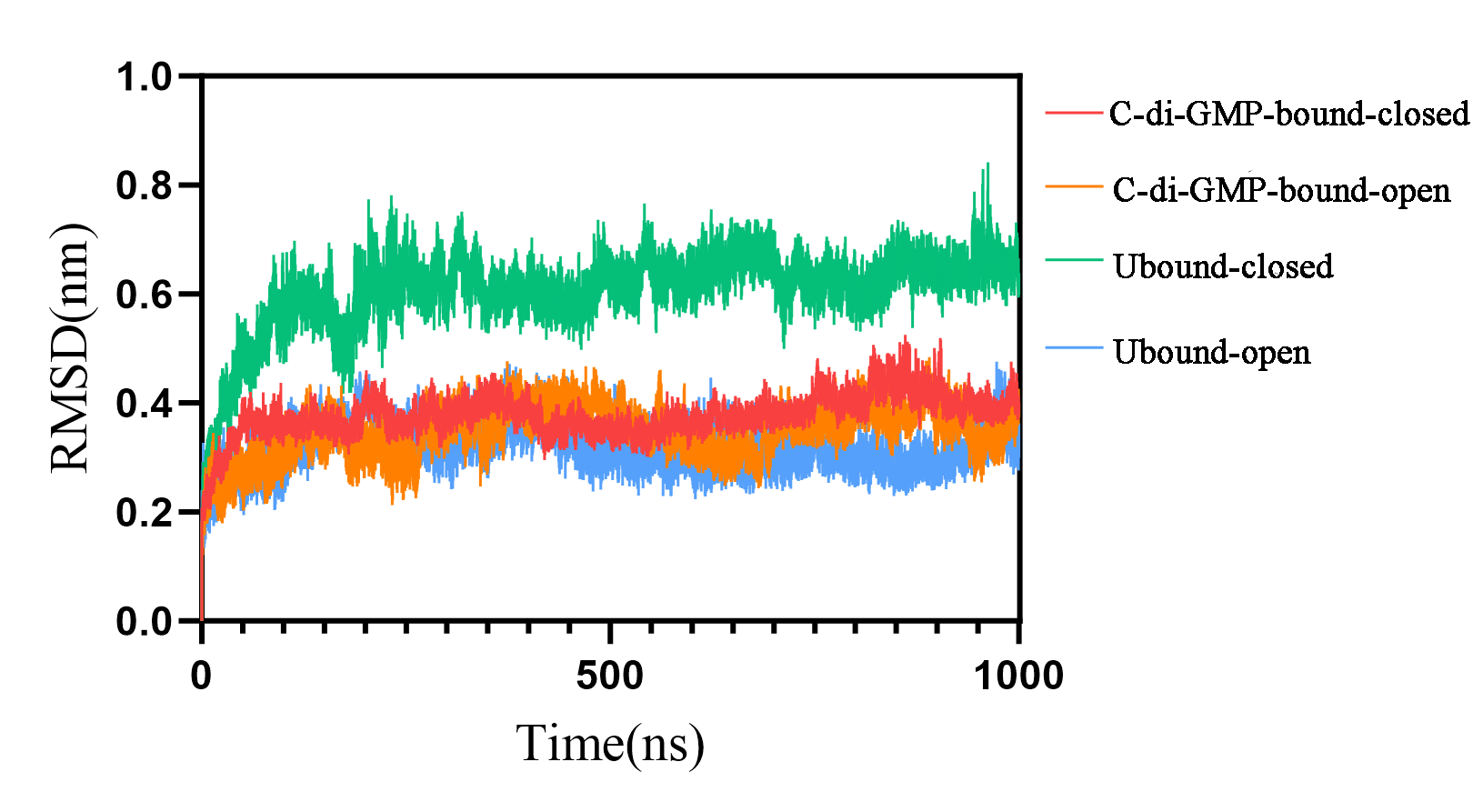

Supplement: Supplementary 1 — Figs. S1 to S13 [file csbj.0133.f1.zip › Supplementary Information-Fig/Figure S6.tif]

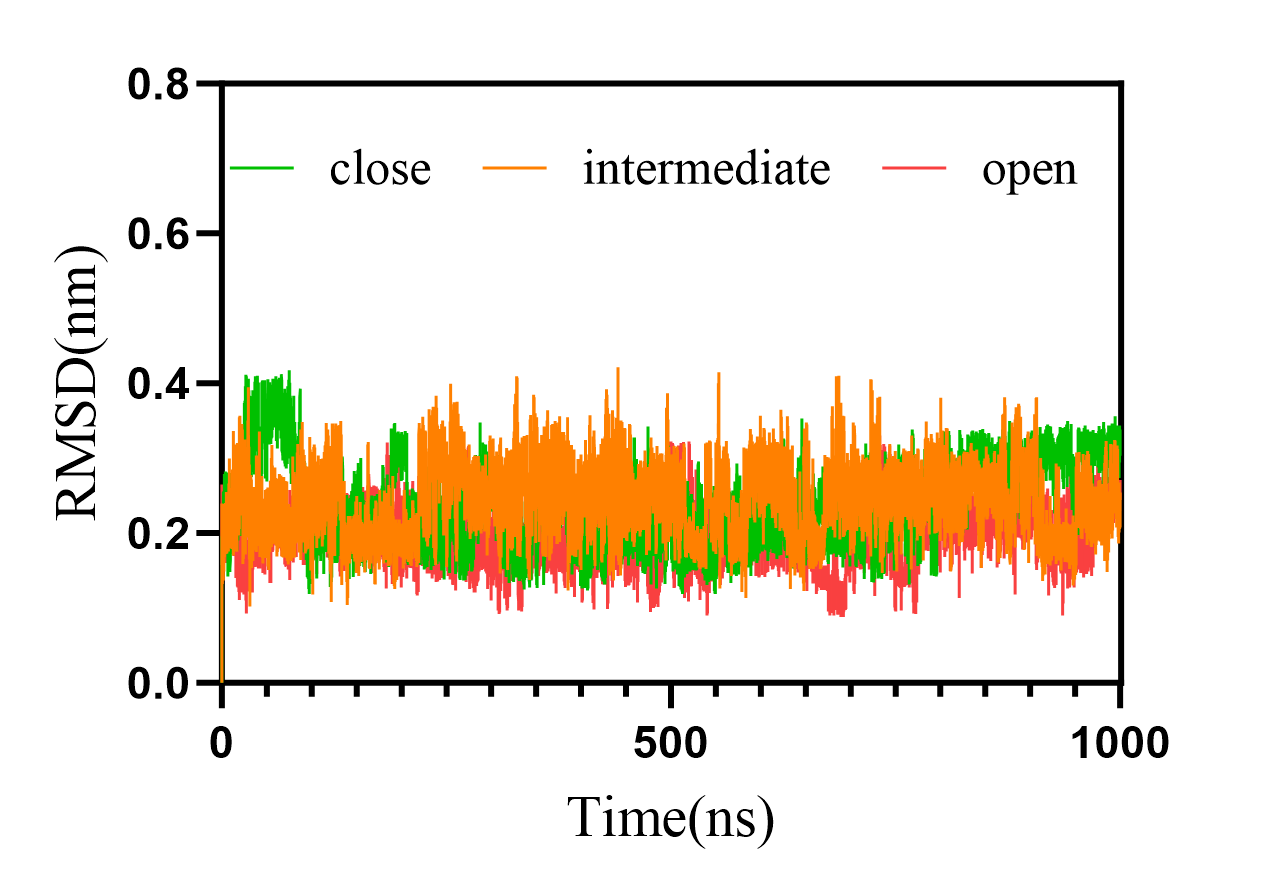

Supplement: Supplementary 1 — Figs. S1 to S13 [file csbj.0133.f1.zip › Supplementary Information-Fig/Figure S7.tif]
